# A systematic review on the use of quantitative imaging to detect cancer therapy adverse effects in normal-appearing brain tissue

**DOI:** 10.1007/s10334-021-00985-2

**Published:** 2021-12-17

**Authors:** Jan Petr, Louise Hogeboom, Pavel Nikulin, Evita Wiegers, Gwen Schroyen, Jesper Kallehauge, Marek Chmelík, Patricia Clement, Ruben E. Nechifor, Liviu-Andrei Fodor, Philip C. De Witt Hamer, Frederik Barkhof, Cyril Pernet, Maarten Lequin, Sabine Deprez, Radim Jančálek, Henk J. M. M. Mutsaerts, Francesca B. Pizzini, Kyrre E. Emblem, Vera C. Keil

**Affiliations:** 1 Helmholtz-Zentrum Dresden-Rossendorf, Institute of Radiopharmaceutical Cancer Research, Dresden, Germany; 2 Department of Radiology and Nuclear Medicine, Amsterdam UMC, Amsterdam Neuroscience, Amsterdam, The Netherlands; 3 Department of Radiology, University Medical Center Utrecht, Utrecht, The Netherlands; 4 Department of Imaging and Pathology, KU Leuven, Leuven, Belgium; 5 Danish Center for Particle Therapy, Aarhus University Hospital, Aarhus, Denmark; 6 Department of Technical Disciplines in Medicine, Faculty of Health Care, University of Prešov, Prešov, Slovakia; 7 Ghent Institute for Functional and Metabolic Imaging (GIfMI), Ghent University, Ghent, Belgium; 8 International Institute for the Advanced Studies of Psychotherapy and Applied Mental Health, Department of Clinical Psychology and Psychotherapy, Babeș-Bolyai University, Cluj-Napoca, Romania; 9 International Institute for the Advanced Studies of Psychotherapy and Applied Mental Health, Evidence Based Psychological Assessment and Interventions Doctoral School, Babeș-Bolyai University, Cluj-Napoca, Romania; 10 Department of Neurosurgery, Amsterdam UMC, Amsterdam Neuroscience, Amsterdam, The Netherlands; 11 UCL Queen Square Institute of Neurology, University College London, London, UK; 12 Neurobiology Research Unit, Copenhagen University Hospital, Rigshospitalet, Denmark; 13 St. Anne’s University Hospital Brno and Faculty of Medicine, Masaryk University, Brno, Czech Republic; 14 Radiology, Deptartment of Diagnostic and Public Health, Verona University, Verona, Italy; 15 Department of Diagnostic Physics, Division of Radiology and Nuclear Medicine, Oslo University Hospital, Oslo, Norway

**Keywords:** Neuroimaging, Radiotherapy, Chemotherapy, Long-term adverse effects, Cognitive decline

## Abstract

Cancer therapy for both central nervous system (CNS) and non-CNS tumors has been previously associated with transient and long-term cognitive deterioration, commonly referred to as ‘chemo fog’. This therapy-related damage to otherwise normal-appearing brain tissue is reported using post-mortem neuropathological analysis. Although the literature on monitoring therapy effects on structural magnetic resonance imaging (MRI) is well established, such macroscopic structural changes appear relatively late and irreversible. Early quantitative MRI biomarkers of therapy-induced damage would potentially permit taking these treatment side effects into account, paving the way towards a more personalized treatment planning.

This systematic review (PROSPERO number 224196) provides an overview of quantitative tomographic imaging methods, potentially identifying the adverse side effects of cancer therapy in normal-appearing brain tissue. Seventy studies were obtained from the MEDLINE and Web of Science databases. Studies reporting changes in normal-appearing brain tissue using MRI, PET, or SPECT quantitative biomarkers, related to radio-, chemo-, immuno-, or hormone therapy for any kind of solid, cystic, or liquid tumor were included. The main findings of the reviewed studies were summarized, providing also the risk of bias of each study assessed using a modified QUADAS-2 tool. For each imaging method, this review provides the methodological background, and the benefits and shortcomings of each method from the imaging perspective. Finally, a set of recommendations is proposed to support future research.

**Supplementary Information:**

The online version contains supplementary material available at 10.1007/s10334-021-00985-2.

## Introduction

Cancer therapy is associated with a multitude of adverse effects such as cognitive dysfunction and a lower quality of life. These effects most likely have a multifactorial origin and are associated with several components of the treatment. For example, the use of brain radiotherapy (RTx) [1] and various chemotherapy (CTx) agents in both central nervous system (CNS) and non-CNS cancer are associated with normal tissue damage in the brain and subsequent cognitive decline [2, 3]. Despite a co-factoring genetic predisposition [4, 5], the amount of damage and related cognitive deterioration from cancer therapy cannot yet be predicted on an individual level [6], and the cellular mechanisms behind these changes are not well understood. Non-invasive biomarkers of treatment response to cancer therapy in normal-appearing brain tissue (NABT) are thus needed to (i) study the etiology of damage and (ii) obtain early-markers of tissue damage, allowing timely adjustment of the treatment or start of supportive therapy to reduce the risks of associated long-term cognitive and quality-of-life deterioration. Effects on cognition and quality of life become increasingly important issues as new therapies for cancer in the body lead to prolonged survival with cerebral oligometastatic conditions kept stable also due to focused radiotherapy.

Currently, brain imaging research of cancer-therapy adverse effects is primarily focused on changes in brain tissue composition [7] measured with T1-weighted, T2-weighted, FLAIR, and susceptibility-weighted imaging (SWI) sequences. These sequences are conventionally used in the clinical context of tumors, are more studied in the context of adverse effects in normal tissues, and were reviewed several times. More specifically, dose-related changes in gray matter (GM) volume and thickness have been documented a few months after RTx in CNS [8, 9] and CTx in non-CNS cancer [10]. Susceptibility of different brain structures to damage and exact dose tolerance are, however, still unknown [11]. Moreover, cognitive decline after RTx has been associated with microvascular damage [12]. These are hallmarks of vascular-related cognitive changes, typically observed in dementia, and their appearance after RTx points to similarities with neurodegenerative diseases in an accelerated form. Structural changes in white matter (WM), measured with diffusion tensor imaging, have also shown a decrease in fractional anisotropy, potentially reflecting a decrease in fiber density and myelin content, after CTx in breast cancer, associated with cognitive decline [13]. Similar findings after CTx in acute lymphoblastic leukemia [14], and after RTx in CNS tumors [15] are also reported. Since diffusion tensor imaging (DTI) and functional MRI (fMRI) have been used clinically in the context of brain surgery—mapping the networks that need to be kept intact—these sequences have also been reviewed several times for their ability to pick up treatment-induced brain changes [11, 13, 14, 16, 17].

To complement these existing reviews of conventional MRI biomarkers, we set out to review new quantitative imaging biomarkers that are usually not part of the clinical tumor workup. These imaging sequences, measuring physiology and metabolism, may provide early markers of treatment damage, yet, they are usually not considered for evaluation of cancer therapy side effects [18]. The common reasons for this are a lack of insight into the biological specificity of these advanced sequences, the limited available scanning time beyond the standard protocol, and the fact that the complexity of some of these protocols makes them unfit for some of the patients. Insufficient clinical implementation [19], due to difficult acquisition and analysis, often leads to low levels of evidence in this context [20]. To trigger change, more attention needs to be devoted to these techniques, starting by reviewing the current body of literature.

This systematic review explores the current level of knowledge on quantitative imaging techniques for perfusion, metabolism, relaxometry, spectroscopy, advanced diffusion, and susceptibility imaging to evaluate longitudinal alterations in the NABT, in the sense of a visual absence of the tumor, due to both CNS and non-CNS cancer treatment in participants of all sexes and ages. It also assesses the correlation of cross-sectional and longitudinal changes obtained using quantitative measurements with cognitive function changes.

## Methods

This systematic review was registered at PROSPERO number 224196 and followed the PRISMA and PRISMA-P guidelines [21, 22]. The literature search was based on the PICOS tool [23]. The protocols are included in Supplementary Materials.

### Literature search and selection

Briefly, the structure of the search term was

- **Neoplasm**: e.g., liquid tumors, body tumors, CNS tumors, cancer;
- **Normal tissue effect**: e.g., normal brain, adverse effects, cognitive impairment;
- **Neuroimaging**: e.g., MRI, advanced MRI, Positron emission tomography (PET);
- **Cancer therapy**: e.g., RTx, immunotherapy, CTx, hormone therapy.

The exact search strategy is provided in Supplementary Materials and PICOS. Two databases were searched—Medical Literature Analysis and Retrieval System Online (MEDLINE) through PubMed search engine, and Web of Science core collection—on October 13, 2021, without restriction on publication date.

Two reviewers (JP and VK) sorted all publications by titles into four categories and reassessed them in a consensus meeting:

- **A**—Fits most likely with the inclusion/exclusion criteria (Table 1);
- **B**—Reasonable chance that part of data fits with the inclusion/exclusion criteria;
- **C**—Unlikely to contain data on the review subject, but no clear exclusion criteria;
- **D**—Exclusion criteria identified or clearly a different topic.

**Table 1:** Inclusion and exclusion criteria

Inclusion criteria	Exclusion criteria
● Human patients of all ages and sexes ● Longitudinal data before and after treatment OR cross-sectional design with an appropriate control group or a different treatment arm that allows evaluating the treatment effects after treatment ● Present or past neoplasia anywhere in the body ● Local or systemic treatment by RTx, CTx, or hormone therapy ● Advanced quantitative computed tomography, MRI (i.e. ASL, CEST, DCE, DSC, DKI, IVIM, mcDESPOT, MRS, MRSI, MWI, NODDI, qMT, QSM, relaxometry, VERDICT), PET/SPECT (DOPA, FDG, TRODAT, HMPAO, TSPO, 15O-H2O) imaging in the brain ● Dedicated description of imaging findings in NABT as a reaction to cancer treatment	● Not an original research article (e.g. review, conference proceedings, case study, protocol) ● Preclinical research ● Animal research ● Language other than English ● Study containing results only from structural MRI (e.g. DWI, DTI, brain volumetry, T1w, T2w, FLAIR, STI, SWI) or functional MRI ● Findings are reported in normal tissue only in close vicinity of the tumor

Publications that fulfill all inclusion criteria and neither exclusion criteria were considered. Publications containing additional results to advanced imaging of normal tissue—e.g. extra structural imaging, tumor imaging—were considered

**Fig. 1:**
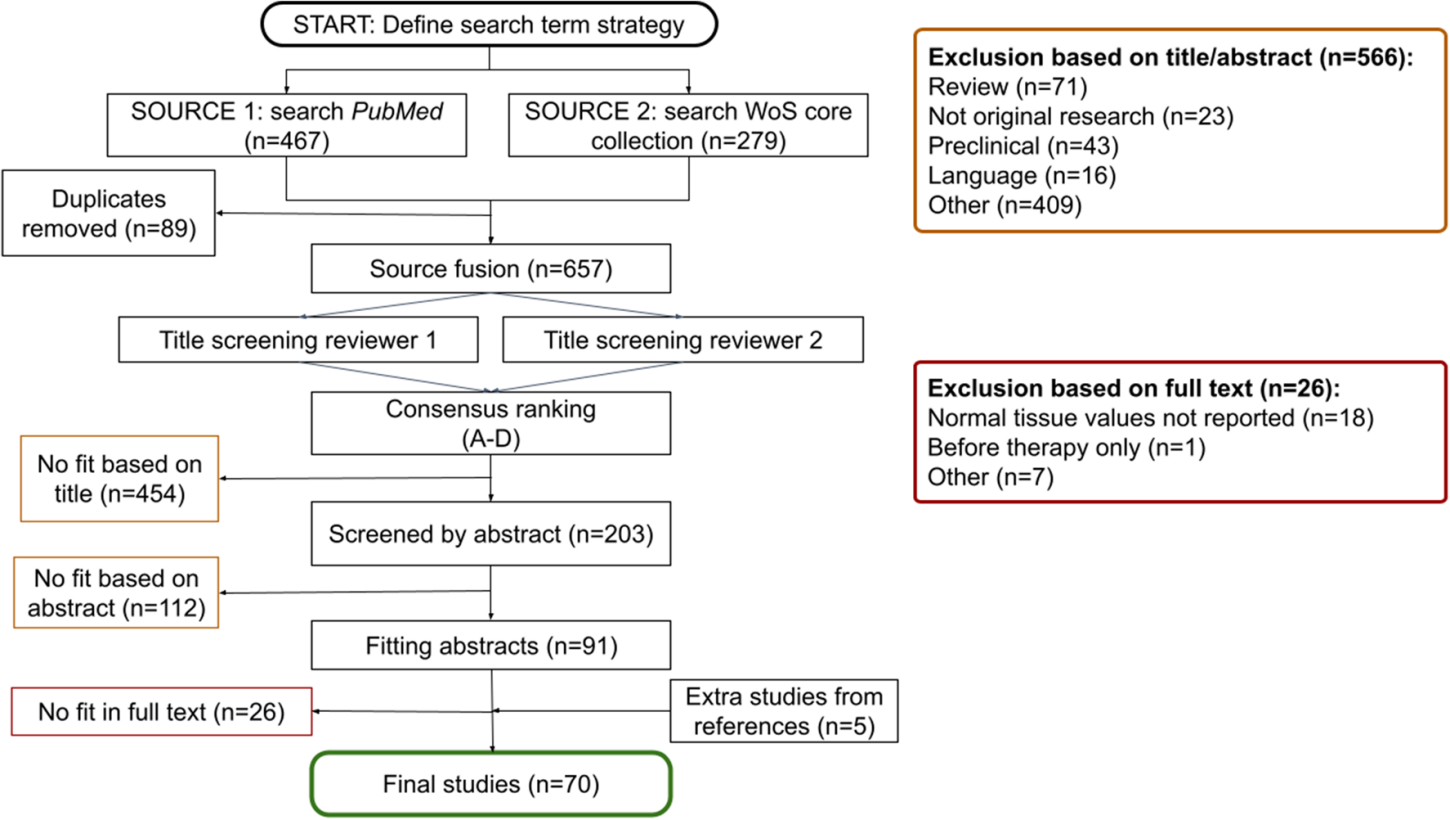
Workflow of the study search and selection. Two databases were searched using the predefined search strategy—MEDLINE, Medical Literature Analysis and Retrieval System Online (PubMed), and Web of Science database (WoS). After excluding the duplicates the studies were initially screened by title (excluding *n* = 446 studies), abstract (excluding *n* = 96 studies), and full text (excluding *n* = 25 studies), leaving 60 studies. Six more studies were discovered by screening the reference sections of selected studies

Studies in categories D were excluded based on title only. Studies in groups A, B, C were reassessed for compliance with the inclusion and exclusion criteria by screening their abstracts. The full text was consulted in unclear cases (Fig. 1).

### Critical appraisal

To assess the quality of the included studies, the QUADAS-2 tool [24] was modified to assess observational imaging studies (Table 2). This modified QUADAS-2 tool represents a list of questions from five different domains that represent the “risk of bias” associated with each study. Complete details and guidelines for scoring can be found in the Supplementary material.

**Table 2:** Modified QUADAS-2 tool

QUADAS domain	Risk of bias questions
Domain 1: Patient selection	Study design (retrospective vs prospective) Patient selection Clear description of selection criteria Case description
Domain 2: Index test	Image acquisition description Image analysis description Region of interest and data extraction
Doman 3: Reference test	Tumor classification quality Treatment details description
Domain 4: Flow and timing	Timing of treatment and imaging described
Domain 5: Data analysis, processing, and reporting	Withdrawals and exclusions explained Statistical tests reported and sufficient Data availability

The quality of the reporting for each study was assessed in five domains. For each domain, one to three aspects were scored as having low, medium, or high risk of bias. A detailed explanation is provided in Supplementary Materials

### Data extraction

Bibliography, patient demographics, tumor type and grade, therapy type (RTx, CTx, hormone, immunotherapy, or other therapies) and doses, imaging modality and sequence, modified QUADAS-2 score, timeline of imaging, a summary of main imaging findings, and association with cognition and QoL were extracted from each study. The following notation was used for reporting the timing of the adverse effect:

- **TS**—time of surgery/biopsy before the start of treatment
- **T0**—*baseline measurement* before the start of the RCTx/immuno/hormone therapy
- **T1**—*acute effects* during therapy or until 1 month after the therapy start
- **T2**—*early delayed effects* at 1–6 months after the therapy end
- **T3**—*late-delayed effects* at more than 6 months after the therapy end

Co-authors were assigned so that each modality would be reviewed by the same person—DSC (KE), DCE (JK), ASL (LH), DKI (JK), relaxometry (RN), QSM/qMT/NODDI (VK), PET/SPECT (PN), MRS (MC, RJ, GS, EW). For papers with more than one modality, the imaging data were reviewed separately for each modality by the relevant reviewers and non-imaging data were reviewed by only one of the reviewers. Non-imaging data from pediatric studies (ML, EW) and breast cancer studies (SD, GS) were extracted separately. Cognitive tests and QoL were assessed separately by (LAF, FBP, and CP). All entries were verified by one co-author (JP). The studies were reviewed, scored, and summarized focusing on the level of evidence for finding tissue damage in different methods and methodological issues that could explain heterogeneous findings. Also, the correlation of the imaging changes with changes revealed using cognitive testing was reviewed. QUADAS scores and extracted data were filled in an online Google-Sheet to allow all co-authors to fill the data in parallel and prevent conflicting entries, especially for publications containing results for multiple sequences.

## Results

The search resulted in 746 records of which 70 studies were eligible for review (Fig. 1).

### Perfusion imaging

Animal studies have shown that acute vascular injury after radiotherapy leads to vessel dilation and damage of the vessel endothelium [25, 26]. This is followed by thrombi causing microvascular occlusions [27] as a possible trigger for WM-lesion formation [28]. However, a potentially more sensitive marker of microvascular damage is perfusion imaging, comprising methods with and without exogenous tracers: single-photon emission computed tomography (SPECT), PET, dynamic susceptibility contrast (DSC), dynamic contrast-enhanced (DCE), and arterial spin labeling (ASL) MRI. SPECT and PET use ^99^Tc-HMPAO and ^15^O-H_2_O radiotracers, the latter being the gold standard in cerebral perfusion imaging. However, ^15^O-H_2_O PET is costly and clinically impractical due to the short tracer half-life and the need for arterial blood sampling. More clinically feasible are dynamic gadolinium-based MRI measurements focusing on changes in T2* (DSC) or T1 (DCE) relaxation. The main advantage of DSC is its widespread use [19] with contrast agents available as part of the routine application for most MRI tumor protocols. DSC may, however, underestimate perfusion in the absence of an intact BBB. In contrast, DCE is a widely used technique to assess apparent contrast agent extravasation across BBB but is less sensitive in regions with an intact BBB. The main challenge to both DSC and DCE is the limited reproducibility of arterial input function estimation, rendering the perfusion measurement semi-quantitative only [29]. ASL perfusion MRI addresses most of these issues as it is non-invasive using magnetically labeled blood as an endogenous tracer. ASL is fully quantitative with accuracy and reproducibility [30] comparable to ^15^O-H_2_O-PET [31]. One disadvantage of ASL is its limited SNR and low sensitivity in regions with low CBF such as the white matter.

The review contains 28 studies on perfusion imaging: PET/SPECT (*n* = 4), DSC (*n* = 11), DCE (*n* = 5), ASL (*n* = 9) imaging. Results are summarized in Table 3 and the modified QUADAS-2 scores are in Supplementary Table 1.

**Table 3:** Perfusion results

Study	Modality/MRI field	Number/female (age ± SD y)	Tumor Type/WHO grade	Treatment	Imaging	Main finding
Bian [39]	3T	18/8F (44 ± 12)	HGG III–IV	IMRTx 60 Gy, TMZ 75 mg/m2	DSC	CBV↓ after 1y in corpus callosum vs T0
Fahlström [43]	1.5T	10/?F (59 ± 8)	HGG III–IV	IMRTx 60 Gy, TMZ 75 mg/m2	DSC	CBV↓4.6–6.7%, CBF↓ 5.1–12.5% in GM at 1 m vs < 5 Gy,T0
Fuss [37]	1.5T	25/7F (41 ± ?)	LGG II	RTx 60 Gy, DXM 1.5-8 mg/day	DSC	CBV↓ 30% for > 24 Gy after 1.5-24 m vs T0
Jakubovic [40]	1.5T	19/9F (? ± ?)	Metastasis	SRS 16-24 Gy	DSC	CBV↑ 43%, CBF↑ 34% after 1 m in 5-10 Gy vs T0
Lee [41]	1.5T	22/9F (49 ± ?)	HGG III–IV	RTx 60 Gy	DSC	CBV↓ non-significant vs T0
Nilsen [44]	3T	40/24F (63 ± ?)	Metastasis	SRS 15-21 Gy	DSC	CBF, CBV↓ 5%, vessel caliber↑ 5% after 6–9 m vs T0
Price [38]	3T	3/1F (? ± ?)	LGG II	RTx 54 Gy	DSC	CBV↓ 16–21%, CBF↓ in WM > 33 Gy vs T0
Singh [45]	3T	25/11F (55 ± ?)	recGBM IV	BEV escalating 2–15 mg/kg	DSC	↔ CBF, CBV in WM after 6-12 m vs T0
Stadlbauer [46]	3T	18/8F (54 ± 11)	recGBM IV	BEV 10 mg/kg	DSC	CBV↓ 20–30% after 3 m, 7 m vs T0 and vs no-BEV
		18/6F (53 ± 15)		No BEV		
Weber [42]	1.5T	25/11F (57 ± ?)	Metastasis	SRS 16-20 Gy	DSC	↔ CBF in WM/GM after 1.5-6 m vs T0
Wenz [36]	1.5T	19/3F (40 ± 12)	LGG II	CRTx 60 Gy	DSC	CBV↓ 30% in WM and GM after 6 m vs T0
		13/3F (56 ± 9)	Metastasis	WBRTx 30-40 Gy		
Andre [54]	1.5/3T	18/8F (57 ± ?)	recGBM IV	RTx 60 Gy, TMZ, BEV 7.5-10 mg/kg	pCASL	Acute CBF↓ 13% in MCA region vs T0
Chen [55]	3T	31/31F (47 ± 5)	Breast II–III	DXR 60, CP 600, DTX 100 mg/m	pCASL	CBF↑ 7–12% after 1 m vs T0 and HC
		34/34F (46 ± 4)	HC	No		
Li [53]	3T	21/4F (14 ± ?)	MB IV	RTx 55 Gy, CPT, CP	pCASL	CBF↓ up to 23% after 0.3-15y vs HC and PA group
		18/9F (12 ± ?)	PA I	No		
		64/41F (12 ± ?)	HC	No		
Nudelman [56]	3T	27/27F (50 ± 8)	Breast I–III	RTx 2, DXR 11, CP,DTX, HTx 3	PASL	CBF↑ 7–12% after 1 m vs CTx- and HC group; CBF↑ 35% in CTx + , CBF↑ 13% in CTx- both in precentral gyrus vs HC
		26/26F (52 ± 9)	Breast 0–II	RTx 6, HTx		
		26/26F (48 ± 10)	HC	No		
Nudelman [57]	3T	24/24F (49 ± 8)	Breast I–III	RTx 2, DXR 11, CP, DTX, HTx	PASL	CBF↑ after 1 m and 12 m in CTx + vs T0
		23/23F (59 ± 9)	Breast 0–II	HTx		
Petr [140]	3T	24/? (54 ± 14)	GBM IV	IMRTx 60 Gy, TMZ 75 mg/m2	pCASL	CBF↓ 9.8% in GM after 3 and 6 m vs T0
Petr [9]	3T	44/20F (55 ± 13)	GBM IV	RTx 60 Gy, TMZ 75 mg/m2	pCASL	CBF↓ 10–11% in GM after 3 and 6 m, proton and photon therapy comparable ↓ vs T0
		16/5F (52 ± 16)		Proton 60 Gy, TMZ 75 mg/m2		
Wang [52]	3T	16/? (65 ± 9)	LGG I–II	RTx 36-63 Gy	pCASL	CBF↓ 7–18% after 2-4 m vs T0
		19/? (62 ± 9)	HGG III–IV			CBF↑ 0–18% after 2-4 m vs T0
Weber [42]	1.5T	25/11F (25–73)	Metastases	SRS 16-20 Gy	PASL	↔ CBF WM/GM until 6 m vs T0
Artzi [50]	3T	26/16 (51 ± 12)	HGG III–IV	RTx, BEV	DCE	↔ V_p_ in WM or GM vs T0
		11/? (37 ± 11)	HC	No		
Cao [47]	1.5T	10/1F (45 ± 16)	CNS I–III	3D-CRTx 50-60 Gy	DCE	V_p_↑ 12% after 1 m, K^trans^↑ 52% at w6 of therapy vs T0
Fahlström [48]	1.5T	12/?F (56 ± 11)	HGG III–IV	IMRTx 60 Gy, TMZ 75 mg/m2, BEV	DCE	V_e_↑ 8% after 3 m vs T0
Farjam [49]	1.5T	27/10F (50 ± 12)	CNS I–III	IMRT/3DCRTx 50-60 Gy	DCE	K^trans^ ↑ in hippocampus after 1 m vs T0
Wong [51]	3T	14/9F (?)	Metastases	WBRTx 37.5 Gy	DCE	↔ after 1–5 months vs T0
Gulaldi [33]	SPECT	18/5F (42 ± 13)	Glioma II–IV	RTx 54-64 Gy, CTx	Tc-99 m-HMPAO	CBF↓ 18.5% in < 3 Gy, 22.5% in > 3 Gy after 3-6 m vs T0
Hahn [34]	PET	11/5F (48 ± ?)	CNS I–III	3DCRTx 50-60 Gy	^15^O-H_2_O	CBF↑ 0.6–5.1% in > 10 Gy at 3w vs < 5 Gy, T0; resolved after 6 m vs T0
Taki [32]	SPECT	13/8F (52 ± ?)	CNS, AVM, metastases	SRS 14-25 Gy	Tc-99 m-HMPAO	CBF↓ 4, 7% whole brain after 2w and 3 m in 2-5 Gy vs T0
Vera [35]	SPECT	12/5F (11 ± ?)	AML, AAL	WBRTx 12-18 Gy, HD ara-C 18–36 g/m2	Tc-99 m-HMPAO	Heterogeneous CBF in cortex, cerebellum after 1 m vs T0

**Tumor type**: *AAL* acute lymphoid leukemia, *AML* acute myeloid leukemia, *AVM* arteriovenous malformation, *CNS* central nervous system, *GBM* glioblastoma, *HC* healthy control, *HGG* high-grade glioma, *LGG* low-grade glioma, *MB* medulloblastoma, *PA* pilocytic astrocytoma, *recGBM* recurrent GBM, *WHO* World Health Organization

**Treatment type**: *BEV* bevacizumab, *CTx* chemotherapy, *CPT* cisplatin, *CRTx* conformal RTx, *CP* cyclophosphamide, *DXM* dexamethasone, *DTX* docetaxel, *DXR* doxorubicin, *HTx* hormone therapy, *IMRTx* intensity-modulated RTx, *RTx* radiotherapy, *SRS* stereotactic radiosurgery, *TMZ* temozolomide, *WBRTx* whole-brain RTx

**Imaging**: *ASL* arterial spin labeling, *DSC* dynamic susceptibility contrast, *DCE* dynamic contrast enhanced, *pCASL* pseudo-continuous ASL, *PASL* pulsed ASL

**Findings**: *CBF* cerebral blood flow, *CBV* cerebral blood volume, *K*^*trans*^ exchange rate between V_e_ and V_p_, *V*_*e*_ extravascular, extracellular fractional volume, *GM* gray matter, *V*_*p*_ intravascular blood plasma fractional volume, *MCA* middle cerebral artery, *T0* pre-therapy baseline, *WM* white matter

#### Positron emission and single-photon emission tomographies (PET/SPECT)

Cerebral blood flow (CBF) decreases were observed at low radiation dose: by 4–7% (absolute CBF, 2-5 Gy at 2 weeks and 3 months after stereotactic radiosurgery (SRS) [32]) and by 18.5% (relative CBF normalized to the contralateral region, < 3 Gy at 0.5–6 months after RTx [33]). After high-dose radiation, contradicting results were reported in relative CBF: 22.5% decrease (> 3 Gy, normalization to the contralateral region [33]) and 0.6–5.1% increase (> 10 Gy, normalized to < 5 Gy, 3 weeks after 3D-CRTx) [34]. Qualitatively evaluated regions with hypoperfusion in cortex and cerebellum were reported in acute myeloid and lymphoid leukemia (AML and ALL) patients after CTx, and whole-brain RTx (WBRTx) or total-body RTx [35].

#### Dynamic susceptibility contrast (DSC)

Default DSC assessment provides relative CBF and CBV values only. These estimates can be either semi-quantitative absolute values using normalization to individual integrated arterial input function (AIF), or normalized to a reference region. Using AIF-based quantification in brain-tumor patients, a 30% decrease in CBV was documented 6 months after WBRTx [36], and after fractionated RTx [37], and with reductions of 16–21% in both CBV and CBF at 3 months post-therapy [38]. Up to 40% CBV reduction in the corpus callosum was observed one year after therapy, however, the exact CBV normalization was not mentioned and the regional values were highly variable across time points and brain regions [39]. One of the few contradictory findings showed a large CBV (42.2%) and CBF (33.9%) increase at 1 month after SRS, but only in regions receiving between 5 and 10 Gy [40].

A series of studies used normalization to regions receiving low doses of radiation in brain tumor patients. Normalization to regions < 15 Gy and < 0.5 Gy yielded only non-significant changes 2 months after RTx [41] and 3 months after SRS [42]. Fahlström et al. normalized to a 0–5 Gy region in WM, and reported a CBV decrease of 4.6–6.7% in GM, and CBF decreases of 5.1–12.5% in GM and 3.1–7.4% in WM. Interestingly, changes were not significant when normalizing to a GM region [43]. Transient reductions of 5% in microvascular CBV and CBF were observed in low-dose regions at 6–9 months normalized to < 1 Gy region. A corresponding 5% increase in the average vessel caliber was observed when using a combined gradient-echo spin-echo based readout scheme [44].

Some of the studies above looked at mean regional imaging values at regions defined by their dose from the dose-distribution maps and reported non-significant RT-dose dependence [37, 38, 41], whereas an inverse correlation of CBV and CBF to dose in WM was observed only by Fahlström et al. [43]. While some studies report that CBV plateaus several months after brain RTx and does not resolve for years [37], resolution of perfusion changes in the period of 9–18 months after therapy was also observed, though without adjusting for subgroups at baseline [44].

Mixed results were reported in NABT following anti-angiogenic treatment in glioblastoma patients varying from no perfusion change [45] to a 20–30% CBV decrease 3–7 months after therapy onset compared to both baseline and to bevacizumab-naive patients [46].

#### Dynamic contrast enhanced (DCE)

DCE primarily measures the intravascular blood plasma fractional volume (V_p_), the extravascular, extracellular fractional volume (V_e_), and exchange rate, i.e. BBB leakage, between these two compartments (K^trans^). The reviewed studies reported DCE changes solely in patients with primary brain tumors or metastases receiving cranial radiation.

A V_p_ increase of 4–10% was observed at therapy week 3 of RTx, peaking at 12% 1 month after therapy, resolving to 3-week values at 6 months. K^trans^ increase of 39–52% at week 6 of therapy resolved to pre-therapy values at 6 months, showing correlation to the regional dose and overall irradiated volume [47]. No other DCE studies gave clear results. Transient V_e_ increase by 8% was observed in GM 3 months post-RTx, but K^trans^ and V_e_ at other times had similar magnitudes but were not significant [48]. A longitudinal K^trans^ increase in the hippocampus was claimed one month after RTx, without providing details of the effect size [49]. No changes in V_p_ were observed after RCTx [50], nor in the non-normalized area under the curve (AUC) 6 months after WBRTx [51].

#### Arterial spin labeling (ASL)

ASL did not show CBF changes in NABT at 3 months after SRS in brain metastases patients [42], and unclear findings of both increase and decrease were reported in patients diagnosed with high-grade (HGG) and low-grade gliomas (LGG), respectively [52]. A larger prospective study in glioblastoma patients was, however, in line with the majority of DSC results showing 10–11% CBF decrease 3 and 6 months after photon RCTx at both low and high-dose regions, with a comparable decrease seen in proton-therapy patients alone [9]. CBF decrease was shown in pediatric patients with posterior fossa tumors 7 years after RCTx. CBF in patients with medulloblastoma undergoing RCTx was both lower than in controls and in RCTx-free patients with astrocytoma [53]. Bevacizumab-related acute CBF decrease of 13% was also shown with ASL in glioblastoma patients [54]. In metastasis-free breast cancer patients 1 month after CTx, a CBF increase of 7–12% was shown with respect to both baseline and healthy controls, despite the anti-angiogenic effects of CTx [55], and a similar increase was confirmed by another group compared with both the CTx-naive patients and healthy controls [56, 57].

#### Perfusion imaging discussion

Perfusion decrease after brain RTx was demonstrated by most quantitative perfusion studies, although absolute perfusion values were not always obtained. Using PET (and, to some extent, SPECT) absolute values can only be provided when a properly calibrated acquisition and processing is performed. And DSC and DCE can provide semi-quantitative values only. Therefore, most of the included studies normalized the acquired perfusion results to whole-brain values, a reference anatomical region, or to a low-dose region assuming to be relatively unaffected by the therapy. However, either of these assumptions might be invalid as effects of therapy were shown across the entire brain and in the low-dose regions as well. Such normalization might then mask overall perfusion changes. None of the reviewed studies were able to distinguish between the separate effects of CTx and RTx, although both were independently shown to affect perfusion. Most studies investigated acute or early delayed changes in perfusion and did not investigate late-delayed effects. While a few studies suggested that perfusion changes might resolve after several months, these have to be interpreted with care as the group sizes typically decrease at later time points, and no larger studies with observing longitudinal perfusion deficits after 1 year exist.

Technique repeatability is an important aspect needed for assessing and comparing the significance of the findings between studies. We provide here the literature values of repeatability of techniques in normal tissue for all techniques with at least two reviewed references and a single positive imaging finding. ^99m^Tc-HMPAO SPECT repeatability of absolute flow on rescan was 15.0 ± 1.5% [58]. Very good repeatability of CBV, K^trans^, and CBF, respectively, was reported for DSC (3–11%) [59], DCE (7.7%) [60] and ASL (6.6–14.8%) [30]. These, however, greatly depend on the used parameters, sequence implementation, or models used for quantification and can vary substantially between studies [61, 62].

### Metabolic imaging

Magnetic resonance spectroscopy (MRS) enables the detection of MR signals generated by chemical compounds other than water, such as N-acetyl aspartate (NAA), creatine (Cr), choline (Cho), glutamate, myo-inositol (mI), lactate, and γ-aminobutyric acid. MRS provides an MR spectrum, originating from the nuclei in atoms (e.g. ^1^H), graphically displaying measured signal intensity as a function of their resonance frequency. The relative area under each peak is directly proportional to the tissue concentration of the corresponding nuclei [63]. MRS allows assessing the *in-situ* tissue biochemistry, which may relate to tumor characteristics from different brain-tumor types. The typical excitation and localization scheme is PRESS (point resolved spectroscopy). Increased choline and decreased NAA levels are typically seen in tumors, whereas decreased NAA in NABT indicates decreased neuronal density. Additionally, ^1^H-MRS is highly sensitive to metabolic abnormalities underlying cognitive deficits [64]. Besides MRS, PET is the most used modality to measure brain metabolism. Fluorodeoxyglucose (FDG), 18 kDa translocator protein (TSPO), and tropen derivative (TRODAT) based tracers can be used, respectively, to measure brain glucose uptake, microglia and astrocyte activation, and the presence of dopamine active transporter. CTx is suspected to elevate inflammatory cytokine levels [65], possibly detected by FDG-PET, decrease dopamine release [66], measurable by TRODAT-SPECT, or directly by TSPO-PET through microglia and astrocyte [67], all mechanisms possibly contributing to cognitive decline.

This review contains 39 studies on metabolic imaging: MRS (*n* = 23), FDG-PET (*n* = 6), DOPA-PET (*n* = 1), TSPO-PET (*n* = 1), and TRODAT-SPECT (*n* = 1). Results are summarized in Table 4 and the modified QUADAS-2 scores are presented in Supplementary Table 2.

**Table 4:** Metabolic imaging results

Study	Modality/MRI field	Number/female (age ± SD y)	Tumor Type/WHO grade	Treatment	Imaging	Main finding
Alirezaei [79]	1.5T	10/5F (36 ± ?)	OGD II	3D-CRTx 54 Gy	^1^H-MRS	NAA/Cr↓ 12,18%, Cho/Cr↑ 15,20% at 1,6 m vs T0
Chawla [76]	3T	7/4F (65 ± 11)	Mets, lung	WBRTx 25-40 Gy	^1^H-MRS	Cho/Cr↑ 12%, NAA/Cr↓ 14% in hippocampus; Cho/Cr↑ 11% in corpus callosum, both at 1 m vs T0
Chernow [68]	1.5T	40/18F (61 ± 9)	Metastases	SRS 38 Gy	^1^H-MRS	↔ NAA/Cr, NAA/Cho, Cho/Cr at 1-12 m vs T0
Davidson [83]	1.5T	11/?F (13 ± 3)	ALL, NHL	MTX 6–24 g/m2	^1^H-MRS	Cho↓ 16%; ↔ NAA, Cr, NAA/CR, Cho/Cr, all at 3w-15 m vs HC
		17/?F (11–36)	HC	No		
Davidson [69]	1.5T	17/?F (1–16)	CNS, ALL	Cranial RTx	^1^H-MRS	↔ NAA, Cho, Cr, NAA/Cr, Cho/Cr at 8-10y vs HC
		17/?F (?)	HC	No		
de Ruiter [86]	3T	17/17F (57 ± 5)	Breast II–III	RTx, FEC + CTC, TMX	^1^H-MRS	NAA/Cr↓ 7.8%; ↔ Cho, NAA, Cr, all in left centrum semiovale at 9.5y vs CTx-
		15/15F (58 ± 6)	Breast I	RTx		
Esteve [80]	1.5T	11/3F (44 ± 11)	Glioma II–IV	RTx 60 Gy	^1^H-MRS	Transient NAA↓ 7%, Cho↑ 10%, Naa/Cho↓ 19% at 4 m, Naa/Cr↓ 18% at 1 m, all vs T0
Follin [84]	3T	33/?F (38 ± ?)	ALL	CRTx 18-30 Gy, MTX	^1^H-MRS	NAA/Cr↓ 10% in WM, 4.5% in GM; mI/NAA↑ 16% in WM, all at 34y vs HC
		29/21F (?)	HC	No		
Hattingen [89]	3T	16/5F (50 ± ?)	rec GBM IV	BEV 10 mg/kg	^1^H,^31^P MRS	↔ Cho, NAA, Cr, pCr, pCho, Pi, pH, ATP at 2 m vs T0
Kaminaga [72]	1.5T	20/8F (42–71)	Metastases	WBRTx 40-50 Gy	^1^H-MRS	Cr ↔ , NAA↓, Cho↑ at 3 m vs T0 and T1
Kesler [85]	3T	19/19F (55 ± 8)	Breast I–III	RTx 74%, CTx, TMX42%	^1^H-MRS	↔ NAA, Cr, NAA/Cr; mI↑ 15%, Cho↑ 11%; NAA/Cho↓ 15%, all at ~ 5y (range 1-13y) vs HC
		17/17F (56 ± 9)	HC	No		
Lee [73]	1.5T	10/5F (55 ± 16)	LGG II	IMRTx 60 Gy, TMZ	^1^H-MRS	Cho/Cr↑14% at 2 m, NAA/Cr↓5–7% > 25 Gy at 2-6 m, vs T0
Pospisil [77]	3T	10/4F (60 ± 7)	Metastases	WBRTx 30 Gy, CTx 50%	^1^H-MRS	NAA↓ 16% in hippocampus at 4 m vs T0
Pospisil [78]	3 T	18/7F (58 ± 8)	Metastases	WBRTx 30 Gy, CTx 75%	^1^H-MRS	NAA↓13%,Cr↓8%, NAA/Cr↓5% in hippocamp. at 4 m vs T0
Rutkowski [71]	2T	43/15F (40 ± ?)	Glioma II–III	3D-CRTx 60 Gy	^1^H-MRS	NAA/Cr↓ 27–31%, Cho/Cr↑ 4–14% in < 6 Gy & 21-39 Gy, all at 9-12 m vs HC and vs T0
		30/15F (29 ± 7)	HC	No		
Rueckriegel [74]	3T	24/?F (14 ± 5)	MB IV	RTx 16-32 Gy, CTx	^1^H-MRS	NAA↓ 9% in GM, 12% in WM at 3.4y in MB vs HC. NAA↓ 4% in GM at 2.1y in PA vs HC. ↔ Cr, Cho
		15/?F (11 ± 4)	PA I	No		
		43/?F (14 ± 5)	HC	No		
Stouten-Kemperman [87]	3T	19/19F (56 ± 6)	Breast I–III	RTx, FEC + CTC, TMX	^1^H-MRS	NAA/Cr↓ 6% at left centrum semiovale at 12y in high-CTx + vs low-CTx + , CTx-, and HC
		24/24F (60 ± 6)	Breast I–III	RTx, FEC, TMX		
		15/15F (58 ± 6)	Breast I	RTx, TMX 7%		
		20/20F (60 ± 5)	HC	No		
Stadlbauer [46]	3T	18/8F (54 ± 11)	rec GBM IV	BEV 10 mg/kg	^1^H-MRS	↔ Cr, Cho, NAA in BEV + and BEV- at 3-7 m vs T0
		18/6F (53 ± 15)	rec GBM IV	no BEV		
Sundgren [81]	1.5T	11/1F (44 ± 16)	Intrcran I–II	3D-CRTx 50-60 Gy	^1^H-MRS	NAA/Cr↓ 9–21%, Cho/Cr↓ 9%-23% at T1 till 6 m vs T0
Tong [88]	3T	24/24F (43 ± 4)	Breast I–II	RTx 29%, DTX/PTX + CP, HTx 54%	^1^H-MRS	NAA↓ 1–4% in posterior cingulate gyrus and dorsal thalamus at 2-3w after vs T0, ↔ NAA in CTx- vs T0
		20/20F (42 ± 4)	Breast 0–I	RTx 20%, HTx 50%		
Usenius [70]	1.5T	8/5F (48 ± 11)	CNS II	RTx 57 Gy	^1^H-MRS	NAA/Cr↓ 25% > 59 Gy, ↔ NAA/Cr < 59 Gy, ↔ Cho/Cr, all at 6 m-13y vs HC
		5/?F (?)	HC	No		
Virta [82]	1.5T	9/5F (54 ± 5)	glioma II–IV	RTx 62 Gy, CTx 30%	^1^H-MRS	NAA/Cr↓ 24% between T1 and 6 m-10.5y vs HC
		9/5F (50 ± ?)	HC	No		
Waldrop [75]	1.5T	70/?F (11 ± 5)	CNS	RTx 26-54 Gy, CTx	1H-MRS	NAA/Cr↓ 15% (CTx + ↓ 22%, CTx- ↓ 12%) vs HC
		11/?F (10 ± 3)	HC	No		
Carideo [97]	PET	48/22F (45 ± 13)	Glioma II–IV	RTx ?%, CTx-	18F-DOPA	DOPA ↑ 21% in females at > 1 m vs during TMZ, ↔ DOPA in males, ↔ DOPA at > 1 m vs T0
		57/21F (50 ± 13)		RTx ?%, TMZ		
		50/26F (54 ± 12)		RTx ?%, during TMZ		
Hahn [34]	PET	11/5F (48 ± ?)	CNS I–III	3D-CRTx 50-60 Gy	FDG	FDG↓ 1.1–6.5% at 3w-6 m vs T0
Pomykala [90]	PET	23/23F (51 ± 10)	Breast I–III	RTx 87%, HTx 61%, CTx 35%	FDG	↔ FDG at 12 m vs T1
		10/10F (56 ± 7)	Breast 0–II	RTx 80%, HTx 60%		
Ponto [92]	PET	10/10F (74 ± 4)	Breast I–III	CP + MTX, TMX 50%	FDG	Regional FDG difference both positive and negative vs HC
		10/10F (75 ± 10)	HC	No		
Schroyen [94]	PET	15/15F (51 ± 8)	Breast II–III	CTx (E + CP + PTX)	TSPO	V_T_ ↑ 9% in left and right occipital- and left parietal lobes in CTx + vs HC. V_T_ ↑ 11% in right parietal lobe in CTx + vs CTx-
		15/15F (49 ± 6)	Brest 0–III	No		
		15/15F (44 ± 10)	HC	No		
Shrot [95]	PET	14/3F (3–17)	NHL	CTx	FDG	Regional FDG ↓3% and ↑3% per year vs T0
Silverman [91]	PET	11/11F (52 ± 5)	Breast	CTx, TMX	FDG	FDG↓ 7–8% in lentiform nucleus in CTx + TMX vs CTx + only. ↔ FDG between CTx + , CTx-, and HC at 7y
		5/5F (48 ± 6)	Breast	CTx		
		5/5F (53 ± 4)	Breast	No		
		3/3F (58 ± 7)	HC	No		
Sorokin [96]	PET	21/?F (63 ± 11)	NHL	CTx	FDG	FDG↓ 20% in cortex at 5 m vs T0
Vitor [93]	SPECT	28/28F (50 ± 9)	Breast I–III	RTx 88%, DXR, CP	Tc-99 m TRODAT	DAT↓ 20–23% at average 30 m vs HC
		22/22F (50 ± 7)	HC	No		

**Tumor type**: *AAL* acute lymphoid leukemia, *CNS* central nervous system, *GBM* glioblastoma, *HC* healthy control, *LGG* low-grade glioma, *MB* medulloblastoma, *NHL* non-Hodgkin lymphoma, *OGD* oligodendroglioma, *PA* pilocytic astrocytoma, *recGBM* recurrent GBM, *WHO* World Health Organization

**Treatment type:** *FEC* 5 fluorouracil, epirubicin, and CP, *BEV* bevacizumab, *CTx* chemotherapy, *CPT* cisplatin, *CRTx* conformal RTx, *CTC* CP, thiotepa, and carboplatin, *CP* cyclophosphamide, *DXM* dexamethasone, *DTX* docetaxel, *DXR* doxorubicin, *HTx* hormone therapy, *IMRTx* intensity-modulated RTx, *MTX* methotrexate, *PTX* paclitaxel, *RTx* radiotherapy, *SRS* stereotactic radiosurgery, *TMX* tamoxifen, *TMZ* temozolomide, *WBRTx* whole-brain RTx

**Imaging:** *DAT* dopamine active transport, *MRS* magnetic resonance spectroscopy, *FDG* fluoro-deoxy-glucose, *TRODAT* tropane derivative radiopharmaceutical for imaging of DAT binding, *TSPO* translocator protein

**Findings:** *ATP* adenosine triphosphate, *Cho* choline, *Cr* creatine, *GM* gray matter, *Pi* inorganic phosphate, *mI* myo-inositol, *NAA* N-acetyl aspartate, *pCr* phospho-creatinine, *pCho* phospho-choline, *T0* pre-therapy baseline, *V*_*T*_ total distribution volume, *WM* white matter

#### Magnetic resonance spectroscopy (MRS)

Only two studies did not find significant changes in NAA, choline, or creatinine, or their ratios in NABT following cranial RTx—in brain metastasis patients after 1 to 12 months [68], and in CNS and leukemia patients after 10 and 8 years [69]. Almost all others reported a decrease in NAA, Cr, or NAA/Cr, or an increase in Cho or Cho/Cr. While one of the first cross-sectional studies on ^1^H-MRS changes in NABT in ALL subjects showed changes only at dose > 59 Gy [70], later studies in gliomas patients found changes even in doses below 6 Gy, compared with healthy controls and pre-therapy baseline [71], or as early as 1 week after WBRTx [72]. Limited evidence of dose effect was available comparing low and high-dose regions after RCTx [73], and patients with and without RTx [74]. Combined RCTx seemed to worsen the effect in pediatric brain tumor patients in comparison with RTx only [75]. While these studies mainly focused on regions contralateral to the tumor, metabolic changes were also reported in specific anatomical regions irrespective of tumor location. Hippocampal metabolic changes were reported 1 month [76] and 4 months after WBRTx [77, 78]. Additionally, a progressive worsening was reported between week 4 of 3D-CRTx until 6 months after, with NAA/Cr in the corpus callosum further decreased from 10 to up to 18% [79]. However, the decrease of NAA/Cho by 19% 4 months after RTx resolved to pre-therapeutic levels another 4 months later in another study [80]. Only two studies were in partial conflict with the results above. A decrease of both NAA/Cr and Cho/Cr in NABT was reported until 6 months after 3D-CRTx [81]. A lower Cho/Cr, and higher NAA/Cr and NAA/Cho in normal-appearing WM (NAWM) after RTx was reported with respect to healthy controls [82].

Besides a single pediatric study with ALL patients, reporting decreased choline levels [83], metabolic changes following CTx treatment of non-CNS tumors were mostly consistent with brain RT-related changes. ALL patients receiving methotrexate and cranial RTx at 34 years after the diagnosis showed a decrease of NAA/Cr and an increase of myo-inositol (mI) in deep WM and parieto-occipital GM [84]. Metabolic changes were also shown cross-sectionally in four studies including breast cancer patients undergoing locoregional chest RCTx and hormone therapy. Higher mI and choline, and lower NAA/Cho were reported 5 years after therapy compared with healthy controls [85]. But NAA/Cr decrease was shown also after 10 years in comparison with CTx-naïve patients treated with RTx only [86], or after 12 years in comparison with healthy controls or patients without chemo- or hormone-therapy [87]. Lastly, NAA and Cr decrease in the posterior cingulate gyrus and dorsal thalamus at 2–3 weeks after therapy was shown compared with baseline in patients with RCTx but not in patients with hormone therapy only [88].

Finally, the influence of anti-angiogenic therapy on NABT was studied longitudinally in recurrent glioblastoma in two studies after 3 and 7 months [46] and after 2 months [89]. Neither study found significant changes in either NAA, Cr, or Cho, and the second study also reported a lack of changes in ^31^P results of pH, adenosine triphosphate, phosphomonoesters, phosphodiesters, phosphocreatine, and inorganic phosphate.

#### PET and SPECT metabolic imaging

Similar to the perfusion findings, the metabolic measurements were mostly normalized to whole brain or low-dose region values. Consistent with other findings after brain RTx, glucose uptake decreased 3 weeks and 6 months post-treatment in the region > 10 Gy when normalized to < 5 Gy region [34]. No clear findings were obtained with FDG-PET in three studies with breast cancer patients. No uptake differences between baseline and 1y after chemo and hormone therapy were observed when normalizing to the global mean [90]. No differences were observed between CTx and control groups 7 years after the therapy, although a 7–8% decrease was observed in the lentiform nucleus between the hormone and CTx and hormone-therapy only groups when normalized to the all-ROIs mean [91]. Higher uptake than in controls was observed 16 years after chemo and hormone therapy in postcentral gyrus and corpus callosum after a global normalization, but the effect was opposite in frontal gyri, substantia nigra, and brainstem [92]. Breast cancer survivors reporting chemo-fog were examined using ^99^Tc-TRODAT-1 SPECT measuring dopamine transport, reporting a 20–23% decrease in the putamen, caudate, and striatum [93], and TSPO-PET showed signs of neuroinflammation in the occipital and parietal lobes both compared to non-treated patients and a healthy group 4 weeks after (6 months of) CTx [94]. Finally, two studies reported on CTx effects in patients with non-Hodgkin lymphoma. A glucose uptake increase was measured in the parietal and cingulate cortex, and a decrease in basal ganglia, brainstem, and thalamus when normalized to whole-brain activity [95]. In one of the two studies that used non-normalized standard uptake values (SUV), mean cortical glucose uptake decreased 20% between pre-therapy and 1 to 14 months post-therapy [96]. The other study reported a brief 21% increase of ^18^F-DOPA SUV during TMZ therapy in female glioma patients [97].

#### Metabolic imaging discussion

Absolute quantification of ^1^H-MRS signals requires normalization to the concentration of an internal or external reference metabolite, of which stability is of utmost importance. In the reviewed studies, typically an internal reference was used, either the tissue-water concentration or an individual metabolite (e.g. creatinine). Notably, several studies reported creatinine changes in NABT after cancer treatment, which may confound metabolic ratios. Moreover, despite that most studies with non-CNS tumors placed the MRS ROI at an anatomical location in deep WM, the majority of studies with CNS tumors specified the location as contralateral to the tumor, without providing the detailed anatomical location of the tumor, the distance from the tumor, or WM and GM content. This may have led to signal contamination with tumor-related changes, and the mixing of signals from different tissue types. Consistent voxel prescription and anatomical landmarks are recommended to improve inter-patient and inter-study reproducibility and to comply with the recent consensus on MRS reporting standards [98]. Additionally, a further consensus recommends the sLASER sequence for 3T MRI [99], while PRESS was used in the majority of the reviewed studies. Another main shortcoming was that hormone therapy with tamoxifen is known to influence brain metabolites [100] and most studies reviewed here were not designed to easily disentangle the effect of different chemotherapeutics and tamoxifen. Finally, more attention should be given to other metabolites than NAA, choline, and creatinine since changes related to cognitive performance were reported in other metabolites, e.g., mI.

Reproducibility of MRS at 3 T with PRESS sequence showed a median between-session coefficient of variation for the five major metabolites — NAA, tCr, Glu, tCho, and Ins—between 2.5 and 5.3% [101], and similar values below 5% were obtained at 7T with semi-LASER sequence [102]. In oncological patients, the test–retest coefficient of variation in ^18^F-FDG PET was estimated to be 10.0 ± 3.1% for tumor SUV mean [103] and can be expected to be lower in normal brain tissue, although no clinical studies are available to confirm this assumption.

The main concern for PET/SPECT was that its results were normalized to the whole brain or a region receiving a low dose of radiation. For treatments that are likely to affect the entire brain—e.g., chemo- or hormone therapy—this may have masked true metabolic changes. The use of SUV might be a better solution for future studies if additional care is taken to alleviate the shortcomings of SUV [104].

### Advanced diffusion, susceptibility, and relaxation imaging

Diffusion-weighted imaging (DWI) and diffusion tensor imaging (DTI) models are most commonly used for diffusion imaging of WM in the clinical context. While sensitive to changes in the tissue microstructure, specificity to individual microstructural features appears to be insufficient. Two other models—neurite orientation dispersion and density imaging (NODDI) [105] and diffusion kurtosis imaging (DKI) [106]—are tackling this drawback by analyzing multi-shell diffusion data with b-values above 1500 s/mm^2^ [107]. NODDI attempts to disentangle the microstructural complexity of nerve fibers in vivo by delivering multi-compartmental maps of neurite orientation dispersion index (ODI) and neurite density index (NDI). DKI delivers a unitless apparent kurtosis factor K describing the share of non-Gaussian water movement. A clinical application for NODDI in oncological brain MRI is the quantification of white matter structural loss due to treatment, as neural density is expected to decrease due to brain radiation. DKI reveals tissue inhomogeneities, which can be used to evaluate tumor and NABT response to treatment through its presumed sensitivity to cell density and heterogeneity [108], such as in inflammation or apoptosis. However, within the limited scanning time, it is important to consider what features are of interest as multi-shell sequences (e.g. NODDI) typically have longer scanning times.

Quantitative susceptibility mapping (QSM) and myelin water imaging (MWI) are two techniques based on the quantification of T2* relaxation. QSM modifies susceptibility-weighted imaging (SWI) to detect weak susceptibility changes and generates maps of quantified susceptibility parameters, for example, based on multi-echo acquisitions [109]. QSM, or directly T2* mapping, can be used to quantitatively assess (micro)hemorrhages and iron deposition from blood, as an association between microbleed incidence with RTx dose and cognitive decline was previously shown on the qualitative evaluation of SWI [12]. MWI is based on deconvolution of myelin-water components of T2-decay curves [110, 111], and is ideal for imaging de- and remyelination as well as gliosis. While atrophy after cancer therapy can be identified in T1-weighted images, T1-time mapping can possibly identify more subtle changes in tissue density, gliosis, or edema. Finally, quantitative magnetization transfer (qMT) is a technique that can be used to estimate the amount of magnetization transfer between the semi-solid macromolecular pool—including myelin—and the free water pool but needs additional multi-frequency acquisition to generate the MT spectrum [112].

This review contains 12 studies on advanced diffusion, susceptibility, and relaxation imaging: NODDI (*n* = 3), DKI (*n* = 7), qMT (*n* = 1), QSM (*n* = 2), MWI (*n* = 1), and relaxometry (*n* = 2). Results are summarized in Table 5 and the modified QUADAS-2 scores are in Supplementary Table 3.

**Table 5:** Advanced diffusion, susceptibility, and relaxation imaging results

Study	Modality/ MRI field	Number/female (age ± SD y)	Tumor Type/WHO grade	Treatment	Imaging	Main finding
Chen [120]	3T	14/14F (66 ± 5)	Breast I-III	CTx	QSM	↔ QSM in caudate, globus pallidus, putamen, thalamus at 1 m, 5 m vs HC
		13/13F (68 ± 6)	HC	No		
Cushing [121]	3T	20/?F (?)	GBM IV	RTx 60 Gy, TMZ, ASCH	QSM	↔ QSM in ASCH + and ASCH- group at T1 vs T0
Mehrabian [122]	3T	16/3F (55 ± ?)	GBM IV	RTx 60 Gy, TMZ	qMT	↔ qMT in NAWM at 3-8 m vs T0
Cushing [121]	3T	20/?F (?)	GBM IV	RTx 60 Gy, TMZ, ASCH	T2*	↔ T2* in ASCH + and ASCH- group at T1 vs T0
Steen [123]	1.5T	21/?F (10 ± ?)	CNS I-IV	3D-CRTx 56 Gy, CTx	T1	T1↓ 4.5% in GM and WM at 0.8y vs T0
Billiet [115]	3T	25/25F (44 ± 6)	Breast I-III	RTx 92%, TMX 60%, FEC	MWI, DKI, NODDI	↔ MK, ODI, NDI, Viso, MWF at 3-4y
		14/14F (43 ± 6)	Breast I-II	RTx 79%, TMX 79%		
		15/15F (42 ± 5)	HC	No		
Chakhoyan [114]	3T	23/?F (57 ± ?)	GBM IV	RTx 60 Gy, TMZ	DKI	↔ MK in NAWM contralateral to the tumor vs T0
Romero-Garcia [113]	3T	17/8F (36 ± 10)	Glioma I-IV	RCTx 71%	NODDI	Pre-operative NDI correlated with recovered memory, recovery in NDI correlated with memory scores
Sleurs [119]	3T	33/?F (23 ± 4)	non-CNS	CTx	NODDI, DKI	Viso↑ in central WM, NDI↑ in corticospinal tract vs HC at 9y
		34/?F (22 ± 4)	HC	No		
Stouten-Kemperman [118]	3T	27/0F (43 ± 8)	Testicules	BEP	DKI	Radial kurtosis↑ 9% vs CTx-
		18/0F (48 ± 10)	HC	No		
Tso [117]	3T	20/5F (14 ± 3)	GCT	RCTx (30-54 Gy), Surgery (30%)	DKI	MK↓ in WM, ↔ in GM, at 6.5y (range 1.2–12.2y)
Wu [116]	3T	56/22F (47 ± 11)	NPC II-IV	IMRTx 68-72 Gy, CPT, DTX	DKI	MK↓ 11%,34% in WM; 12%,39% in GM at 1w,12 m vs T0
Wu [126]	3T	54/15F (49 ± 13)	NPC II-IV	IMRTx 68-72 Gy, CPT, DTX	DKI	Axial, radial MK↓ at 1 m in cogn-decline vs non-decline at 2y

**Tumor type**: *CNS* central nervous system, *GBM* glioblastoma, *GCT* germ cell tumor, *HC* healthy control, *NPC* nasopharyngeal carcinoma, *WHO* World Health Organization

**Treatment type:** *FEC* 5 fluorouracil, epirubicin, and CP, *ASCH* ascorbate, *BEP* bleomycin, etoposide, and CPT, *CTx* chemotherapy, *CPT* cisplatin, *CRTx* conformal RTx, *DTX* docetaxel, *IMRTx* intensity-modulated RTx, *RTx* radiotherapy, *TMX* tamoxifen, *TMZ* temozolomide

**Imaging:** *DKI* diffusion kurtosis imaging, *MWI* myelin water imaging, *NODDI* neurite orientation dispersion and density imaging, *qMT* quantitative magnetization transfer, *QSM* quantitative susceptibility mapping

**Findings:** *GM* gray matter, *MK* mean kurtosis, *MWI* myelin water fraction, *NDI* neurite density index, *NAWM* normal-appearing WM, *ODI* orientation dispersion index, *Viso* volume isotropy, *WM* white matter

#### Advanced diffusion imaging

Limited evidence on cancer-therapy-related changes in NABT measured by advanced diffusion models appears to be available. While a baseline correlation of NDI with tumor volume was shown [113], no significant difference in mean kurtosis was reported in WM contralateral to the tumor both in glioblastoma patients [114] and breast cancer patients [115]. The latter study also showed no changes in myelin water fraction, or ODI, NDI, or isotropic volume (Viso) derived from NODDI [115]. However, 11–12% decreases in mean kurtosis in the temporal lobe were measured in GM and WM in patients with nasopharyngeal carcinoma 1 week after RCTx with a further decrease to 34–39% after 1 year [116], and a decrease in WM, but not in GM was also found 1.2–12.2y after pediatric germ cell tumor [117]. An increase of radial kurtosis by 9% in NAWM was reported in testicular cancer survivors after CTx [118]. Last, an elevated Viso in central WM and higher NDI in the corticospinal tract was found in survivors of pediatric sarcoma at 2 to 20 years after CTx [119].

#### Quantitative susceptibility and relaxation mapping

No QSM changes were observed in breast cancer patients at 1 and 5 months after CTx in comparison with a CTx-group [120] nor longitudinally in glioblastoma patients [121]. No longitudinal changes were detected in NAWM of glioblastoma patients using qMT or T2* mapping [122]. Finally, a 4.5% decrease of T1-time was detected both in GM and WM of pediatric brain tumor patients after fractionated RTx at an average of 0.8y follow-up [123].

#### Advanced diffusion, susceptibility, and relaxation imaging discussion

The level of evidence for therapy-related changes in NABT as identified by advanced diffusion techniques (NODDI, DKI) techniques was low as the findings were often non-significant or inconsistent. A potential explanation is that study sample sizes were small and the study designs were mostly cross-sectional. This was similarly true for QSM, qMT, MWI, and T1 and T2* mapping studies. More specifically, only a single T1-mapping study identified treatment-related effects in NABT. Nevertheless, first encouraging results do exist, and result consistency may rise with increasing numbers of studies. It is, therefore, crucial to harmonize study protocols and adhere to the highest quality of acquisition, analysis, and reporting for these techniques.

For NODDI, the inter-session reproducibility of NDI and ODI parameters is below 5% voxel-wise and under 2% regionally. However, ISO has above 40% voxel-wise and above 10% regional repeatability [124]. The repeatability of DKI is not widely established, but the first results report a coefficient of variation between 4% and 5.2% for both RK and MK parameters [125].

### Clinical-imaging association

Several studies investigated either cognition or quality-of-life, together with imaging changes without assessing their association. Either: (a) no cognitive or quality-of-life effects were found [39, 56, 83], (b) no imaging changes were detected [120], (c) the correlations between imaging and clinical findings were not reported [84, 86, 87].

Most studies reported an association of perfusion changes (CBF and CBV decrease, or V_p_, K^trans^ increase) with cognitive or quality-of-life deterioration after brain radio(chemo)therapy. While no correlation of ASL-CBF with full-scale IQ was found in patients with posterior fossa tumors [53], a decrease in ^15^O-H_2_O-PET signal at 6 months post-treatment correlated with retained cognitive function [51]. CBF and CBV decrease and mean vessel caliber increase obtained with DSC after SRS were more pronounced in patients with ECOG (Eastern Cooperative Oncology Group) performance score > 0 on the pre-SRS baseline, hinting that the vasculature of those patients was already impaired at baseline and thus more susceptible to radiation-induced damage [44]. An acute increase of V_p_ in the left temporal and frontal lobes and K^trans^ in the left frontal lobe was correlated with decreased verbal learning rates, and K^trans^ also with decreased recall scores [47]. K^trans^ changes in the hippocampus 1 month post-RTx were correlated with memory function at 6 months (*r* = − 0.95, *p* < 0.0006) and 18 months (*r* = − 0.88, *p* < 0.02) [49]. In breast cancer patients after CTx, ASL-CBF increased and this negatively correlated with a decrease in alerting network score (− 0.452 < *r* < − 0.550) and in the executive control network (0.507 < *r* < 0.680) [55]. An increase in frontal-cortex CBF from ^15^O-H_2_O-PET measurements was associated with symptoms of CTx-induced peripheral neuropathy at 1 month but not at 1 year after therapy [57].

The metabolic changes reported in brain-RTx patients correlated with a decrease in cognitive performance in most studies. NAA/Cr in corpus callosum after 1 month correlated positively with verbal fluency score and visuospatial functioning at 6 months, while Cho/Cr correlated negatively with the memory functioning score [79]. Hippocampal NAA/Cr correlated positively with the visuospatial memory test scores (*r* = 0.66; *p* = 0.008) [78]. And choline levels in NAWM correlated positively with IQ in pediatric patients 10 years after therapy [69]. Brain spectroscopy changes and their correlation to cognitive changes in breast cancer patients were similar to that of patients undergoing brain irradiation. Multifactorial memory score correlated negatively with choline level (*r* = − 0.62, *p* = 0.005) and mI (*r* = − 0.55, *p* = 0.02), but not with NAA/Cho or NAA/mI 5 years after therapy. And no imaging correlations were found with the decreased executive function [85]. Auditory verbal learning scores correlated with NAA in posterior cingulate gyrus (*r*^*2*^ = 0.470–0.5, *p* < 0.01) [88]. Finally, a higher global deficit score (GDS) was weakly associated with inflammation on TSPO-PET in the frontal lobe after CTx in breast cancer [94].

Regional decreases in glucose uptake positively correlated with cognitive function in three studies of breast cancer patients undergoing CTx. Rey-Osterrieth Complex Figure (ROCF) performance was correlated with glucose metabolism in the left inferior frontal gyrus [91] and in the posterior orbital gyrus [92]. Additionally, acute changes in glucose uptake in anterior temporal and medial frontal correlated positively with memory test scores [90]; however, neither of those studies presented treatment-related imaging changes in those regions [90, 91] or both increases and decreases in glucose uptake across different regions were reported [92]. Thus the validity of cognitive-imaging correlations should be investigated in the future in larger studies with multiple comparison corrections. A clear longitudinal decrease in FDG-PET SUV negatively correlated with scores from the Symptom Checklist-90-R 6 months after brain RTx [34].

Finally, association results between diffusion kurtosis and cognitive performance or quality-of-life scores varied between no correlation [118] and a correlation with Montreal Cognitive Assessment (MoCA) score as early as 6 months post-RCTx [116]. A stratification to decline and non-decline groups by MoCA test at 2 years showed higher diffusion kurtosis in WM at 1 and 3 months in the non-decline group, which received 20% lower radiation dose to the brain [126]. One of the rare studies that looked at baseline values reported that memory recover at follow-up correlated both with pre-operative NDI and NDI recovery on follow-up [113]. In survivors of pediatric germ cell tumors, mean kurtosis correlated positively with Karnofsky’s performance score and IQ in several cortical regions, but not on the whole-brain level [117].

Most of the reviewed studies evaluated cognitive and quality-of-life changes using several tests often with several subdomains. However, the correlation with imaging biomarkers was not always corrected for multiple comparisons, possibly invalidating the positive findings. Only nine studies have either described that a proper correction for multiple comparisons was used [44, 51, 79, 90, 117] or examined a correlation with a single test only [69, 91, 116, 126].

#### Clinical-imaging association discussion

Approximately one-third of the reviewed studies contained reports on cognitive or quality-of-life changes, but one-third of those did not find any correlations with imaging findings or did not report them, mostly as no association between the change of cognitive scores and treatment was found. Despite the variable findings between studies, consistent effects of treatment on the NABT for most biomarkers could be found. For the studies including cognition or quality-of-life tests in addition to these imaging biomarkers, decline in these clinical scores consistently correlated with these biomarkers. For example, brain RTx was associated with perfusion decrease and BBB permeability increase, both correlating with cognitive decline. Paradoxically, breast cancer CTx resulted in perfusion increase but was still associated with cognitive decline. In MRS, NAA decreases, and choline increases were observed for both brain RTx and CTx in non-CNS tumors, which in turn correlated to cognitive decrease.

In general, a large number of cognition and quality-of-life tests were calculated per study and multiple comparison correction was not always applied, which can easily result in spurious findings. In combination with a legion of cancer and therapy combinations, different ways to image the brain, and arbitrary assessment intervals, this resulted in a manifold of potential experiments, which are difficult to compare. Therefore, no clear pattern of correlation of imaging findings with a specific cognitive sub-domain or a quality-of-life score could be confirmed. In the future, alignment of the techniques would be beneficial to permit confirmation of these pilot results in larger multi-center studies.

## Discussion

In this systematic review, changes in normal-appearing brain tissue following cancer therapy were summarised, measured using different quantitative imaging techniques in either a cross-sectional or longitudinal design. Taken together, all studies mainly presented evidence on adverse effects of brain RTx in CNS tumors, CTx in tumors outside CNS, and anti-angiogenic treatment in recurrent glioblastomas. Regarding CTx it is currently unknown how inhomogeneities in focal metabolism and vascularization of the brain affect the pharmaco-metabolite distribution and therefore adverse effects as revealed by imaging biomarkers. After cranial RTx, NABT showed perfusion decrease relatively consistently with all modalities after both RTx and combined RCTx, and with only a slight inverse correlation to dose. ^1^H-MRS very consistently reported a decrease of NAA, Cr, and NAA/Cr, and an increase in Cho and Cho/Cr. There was some evidence of correlation to dose, as well as trends towards normalization of changes after several months. However, the effects of dose and anatomical locations of the measured changes are still understudied—mostly because the acquisitions were executed in a single voxel contralateral to the tumor—and future studies with multi-voxel MRS imaging are required to provide more insight. Also, the T1 times in normal tissue only decreased in a single study, and T2*, QSM, or qMT measurements were inconclusive. Only weak evidence for diffusion kurtosis decreases could be found.

In breast cancer patients after CTx, metabolic changes measured with ^1^H-MRS were similar to changes in patients undergoing brain RTx: NAA, Cr, and NAA/Cr decrease, and Cho and Cho/Cr increase. Higher dosage of CTx and inclusion of hormone therapy aggravated these effects. Interestingly, perfusion increases were reported to correlate with cognitive decline. While this was shown both longitudinally and cross-sectionally in two independent studies using ASL, validation is still required to confirm the opposite effects compared with brain RTx. Additionally, no or inconclusive metabolic changes were observed with FDG-PET, which could probably be explained by the use of global normalization of the values. Finally, inconclusive findings from advanced diffusion models were reported in patients receiving CTx for non-CNS tumors: one study reported neurite density increase, while two others reported increase and decrease of diffusion kurtosis—see the summary in Table 6.

**Table 6:** Aggregated findings per sequence and tumor type

Modality	Tumor/Treatment	Studies	Patients	Effects	wsCoV
DSC	Brain/RTx	4/5	123/142	↓ CBV 4.6–30%	2.5–3.5%
	Brain/BEV	½	18/43	↓ CBF 20–30%	
ASL	Breast/CTx	3/3	82/82	↑ CBF 7–12%	6.6–14.8%
	Glioma III–IV/RCTx	4/5	123/142	↓ CBF 10–23%	
DCE	Brain/RCTx	3/5	49/89	↑ V_e_ 8–12%, ↑ K^trans^ 52%	7.7%
Tc-99 m-HMPAO	Brain/RTx + SRS	2/3	31/42	↓ CBF 4–22.5%	15%
^1^H-MRS	Breast/CTx	4/4	79/79	↓NAA, NAA/Cr, or NAA/Cho 1–15%	2.5–5.3%
	Brain/RCTx	12/13	244/284	↓ NAA/Cr 5–31%, NAA/Cho 15–19%, NAA 4–16%; ↑ Cho/Cr 4–20%	
FDG-PET	NHL/CTx	½	21/35	↓ FDG 20%	10%
DKI	Non-CNS/RCTx	¾	130/169	↓ MK 11–34%	4–5.2%

**Tumor type**: *CNS* central nervous system, *NHL* non-Hodgkin lymphoma

**Treatment type:** *BEV* bevacizumab, *CTx* chemotherapy, *RTx* radiotherapy, *RCTx* radiochemotherapy

**Imaging:** *ASL* arterial spin labeling, *DKI* diffusion kurtosis imaging, *DSC* dynamic susceptibility contrast, *DCE* dynamic contrast enhanced, *FDG* fluoro-deoxy-glucose, *MRS* magnetic resonance spectroscopy

**Findings:** *CBF* cerebral blood flow, *CBV* cerebral blood volume, *Cho* choline, *Cr* creatine, *MK* mean kurtosis, *NAA* N-acetyl aspartate, *K*^*trans*^ extravascular and *V*_*e*_ extracellular fractional volume, *wsCoV* within-subject coefficient of variation

Summary results are given for each imaging technique and tumor type when the majority of studies that reported an observed parameter had results in concordance. Single studies, or studies where majority of findings were inconclusive or not in an agreement were not reported. The total number of studies and patients in concordance—out of the total number of studies and subject reporting comparable results—are given. Lastly, the within-subject across session coefficient of variation is provided to provide a reference of repeatability in healthy volunteers

## Recommendations

Based on the finding of this systematic review further quantitative imaging studies should be encouraged, addressing the main study-design weaknesses: inhomogeneity of patient populations in terms of both tumor type and treatment; missing descriptive characteristics of study population and treatment details; missing details on the study timeline; not adhering to the acquisition, processing, and reporting standards of the imaging; and incomplete and thorough reporting of the findings. The modified-QUADAS-2 criteria used to assess the published work in this review can serve as a guideline on how to improve the study design, and how to report the methodology and results in future studies aiming at reducing the risk of bias. The acquisition and processing should be performed and described according to current standards: ASL [127, 128], DSC [129], and MRS [98]. Moreover, normalization to whole-brain or low-dose regions can potentially mask changes as the entire NABT might be affected by the treatment. Using an intact brain region based on previous findings or using the full quantitative potential of the imaging techniques may reduce the risk of not detecting subtle changes. All reported MRI techniques are sensitive to tissue type, providing a different contrast and also different performance characteristics in GM, WM, and CSF. The task of ROI delineation should take this into account, and a detailed description of the delineation should be provided to ease the identification of potential biases. Lastly, including a power analysis with negative findings could help the interpretation of results, if the study population size was large enough to observe significant changes, especially in studies with several treatment subgroups.

A general concern is that many studies were explorative only, with small sample size, and did not show statistically significant changes. Still, interesting trends were reported, and the effect sizes may provide a good starting point for power analyses needed in the preparation of larger future studies. Also for some techniques, only a few studies were available, partly caused by the limited availability of quantitative imaging [19]. The general challenge of these kinds of studies is to separate the effect of treatment from the effect of having a tumor, which puts further pressure on the quality of the study design to minimize confounders. At the same time, further research is needed to separate the individual effects of different types of chemo-, radio-, and immuno-therapies, as well as their synergies. While specific study design, like head-and-neck tumors or proton vs photon radiochemotherapy, can help to separate the tumor from radiation effects or radio- from chemotherapy, respectively, more evidence from pooled large studies will be needed. Several community-driven initiatives, for example, Open Source Initiative for Perfusion Imaging (OSIPI—www.osipi.org) and Glioma MR Imaging 2.0 (GliMR, COST Action CA18206) [20], aim at making knowledge and tools for acquisition and analysis of quantitative MRI widely available to advance the field. Additionally, pooling datasets might be indispensable to investigate subtle therapy effects and their regional variations, and to disentangle the interaction of different treatments, possibly with the help of machine learning methods. To this end, it is necessary to obtain patients’ consent to share their data in a secured, controlled manner, ensuring GDPR compliance [130], and using the standardized BIDS format [131] following existing extensions for e.g. PET [132] or ASL [133]. Finally, it is important to consider that brains already differ in their capacity to cope with therapy-related damage prior to cancer therapy, which can be considered a traumatic event. For example, patients with pre-existent small vessel disease or changes leading to subclinical dementia might have a decreased chance to recover from acute cancer therapy effects on the brain compared with an otherwise healthy individual. Here, lifestyle and age, presence of radio-necrosis, but also the baseline status of the biomarker should be considered as co-factors influencing the biomarker gradient when monitoring cancer therapy effects on the brain.

To summarize the recommendations, we advise that future studies investigating the normal tissue changes following cancer therapy should:

- Provide a complete description of the study population, treatment details and timeline, as well as details of acquisition and processing—the modified-QUADAS-2 criteria in supplementary materials provide guidance for this;
- adhere to the latest recommendations for image acquisition and processing—ASL [127, 128], DSC [129, 134, 135], MRS [98];
- avoid mixed cohorts with inhomogeneous tumor and treatment types;
- correct for multiple comparisons and perform a power analysis to avoid spurious findings and to help interpret negative findings;
- support community efforts for data sharing and collaboration (e.g. OSIPI, BIDS [131], OpenNeuro [136], GliMR [20], or ENBIT – www.enbit.ac.uk) to enable future data pooling in larger studies.

## Limitations

This review has several limitations. First, not all quantitative imaging techniques were represented. For example, blood microcirculation measurement with Intravoxel Incoherent Motion (IVIM) [137], or amino-acid measured glucose metabolism with chemical exchange saturation transfer (CEST) could yield interesting results. However, no such publications matching the inclusion criteria were identified during the search. Second, some of the reviewed studies were primarily focusing on the tumor and NABT changes were reported for the sake of normalization or normalization reproducibility. Thus, the quality of the reporting on these side effects was not always optimal, not being the main focus of the study. However, NABT changes in the vicinity of the tumor were ignored so the reported results were not influenced by tumor growth, infiltration, or radio-necrosis. Still, besides under-reporting, there is no reason to believe that the reported values are structurally biased. Third, we did not compare the results of the quantitative imaging methods with the conventional structural imaging biomarkers. From the dementia field, there is evidence that physiological and metabolic biomarkers can pick up changes in brain pathology earlier than structural biomarkers [138, 139]. We hypothesize that this is also the case for RCTx-induced tissue damage, but this needs to be proven yet. Fourth, the acquisition and evaluation of the advanced MRI techniques reviewed here is typically more complex than is the case for standard MRI. There are large differences between the available sequences [61] and models [62] used for their processing and quantification, leading to substantial instrumental variability [134] of the measured parameters. Currently, there are several initiatives that try to propose standards for acquisition [127], processing [128], reference regions [135], and quantification [129] of the advanced MRI data for different sequences. However, most of these were proposed only after the reviewed studies were published, which further complicates a fair comparison of fidelity of individual results presented here. Finally, it should be acknowledged that group-effect results do not guarantee clinical benefit on the single-patient level. This is expected to be especially challenging for biomarkers with high temporal variability—such as physiological fluctuations in cerebral blood flow—but the effect size may even be too small for existing structural biomarkers; especially in the early stages of treatment-induced damage. Therefore, this needs to be carefully studied in the future.

## Conclusion

Quantitative imaging techniques have the potential to detect cancer therapy-related changes in the NABT and correlate with long-term cognitive decline or quality-of-life deterioration. To date, a relatively small number of studies are published that may provide an estimate of the effect size of treatment. However, the evolution of these quantitative changes in time and dependency on dosing and location is still unclear and needs to be evaluated to establish quantitative imaging as an early marker of tissue damage or a predictor of long-term cognitive outcome. Regardless of its limitations, all reviewed imaging techniques showed promise to measure treatment-related damage in NABT, and most evidence is available for ^1^H-MRS and perfusion imaging with DSC and ASL. However, additional care for correct image acquisition, analysis, and interpretation is highly recommended, especially with respect to reaching sufficient statistical power during study planning. Standardizing methodology and pooling of datasets or adding dedicated imaging sequences to existing large-scale studies might be necessary to address this shortcoming, especially for less common imaging techniques.

## Supplementary Information

Below is the link to the electronic supplementary material.

Supplementary file1 (DOCX 117 KB)

Supplementary file2 (DOCX 24 KB)

Supplementary file3 (DOCX 18 KB)
