# Supplementary material for: A systematic review on the use of quantitative imaging to detect cancer therapy adverse effects in normal-appearing brain tissue
Source: MAGMA. 2021 Dec 17;35(1):163–86. doi: 10.1007/s10334-021-00985-2 (PMC8901489; doi:10.1007/s10334-021-00985-2)
Supplement: Supplementary file 1 — Supplementary file1 (DOCX 117 KB) [file 10334_2021_985_MOESM1_ESM.docx]

**Supplementary Table 1.** Modified QUADAS-2 scoring for the perfusion articles.

|  | |  | QUADAS D1 | | | | D1 | | | D3 | | D4 | D5 | | |
| --- | --- | --- | --- | --- | --- | --- | --- | --- | --- | --- | --- | --- | --- | --- | --- |
| Study | Journal | Acq | Study | Sample | Selection | Case | Acquisition | Analysis | ROI | Diagnosis | Treatment | Flow Timing | Exclusions | Statistics | Data |
| Bian [39] | Sci Rep | DSC | 3 | low | low | low | med | high | low | low | low | low | high | low | high |
| Fahlström [43] | Rad Oncol | DSC | 3 | low | med | high | med | low | med | high | low | low | high | low | high |
| Fuss [37] | IJROBP | DSC | 3 | low | low | low | med | low | med | low | low | low | high | med | high |
| Jakubovic [40] | Tech Canc Res&Treat | DSC | 3 | high | med | low | med | med | low | low | med | low | high | low | high |
| Lee [41] | JMRI | DSC | 2 | high | med | high | low | low | low | med | med | low | high | high | high |
| Nilsen [44] | NeurOncol Adv | DSC | 3 | low | low | low | low | low | low | med | low | low | low | low | high |
| Price [38] | Clin Oncol J | DSC | 3 | high | med | low | low | low | low | low | med | low | high | med | high |
| Singh [45] | AJNR | DSC | 1 | low | low | low | low | med | med | low | low | high | low | med | high |
| Stadlbauer [46] | EJR | DSC | 2 | low | med | high | low | med | low | med | low | low | low | low | high |
| Weber [42] | Invest Radiol | DSC | 3 | high | high | low | low | low | low | low | low | low | low | low | high |
| Wenz [36] | AJR | DSC | 2 | high | med | low | low | low | low | med | med | low | high | low | high |
| Andre [54] | Neuroradiol J | ASL | 1 | low | low | low | low | low | low | med | high | high | low | med | high |
| Chen [36] | Sci Rep | ASL | 4 | high | med | low | med | low | high | med | low | low | low | low | high |
| Li [53] | J Pediatr | ASL | 2 | low | low | low | low | low | low | high | high | low | low | low | high |
| Nudelman [56] | PLOS one | ASL | 4 | low | low | low | low | low | low | med | med | low | low | low | high |
| Nudelman [57] | J Clin Oncol | ASL | 4 | low | low | low | low | low | low | med | med | low | low | low | high |
| Petr [140] | Rad Oncol | ASL | 3 | low | low | high | low | low | low | med | low | low | low | low | high |
| Petr [9] | Rad Oncol | ASL | 4 | low | low | low | low | low | low | med | low | low | low | low | high |
| Wang [52] | EJR | ASL | 3 | low | med | low | low | med | high | med | high | low | high | low | high |
| Weber [42] | Invest Radiol | ASL | 3 | high | high | low | low | low | low | low | low | low | low | low | high |
| Artzi [50] | J Neur-Oncol | DCE | 4 | high | high | high | low | med | low | high | high | high | high | med | low |
| Cao [47] | Clin Canc Res | DCE | 3 | high | med | low | low | med | med | med | med | low | high | med | high |
| Fahlström [48] | Acta Radiol Open | DCE | 3 | high | low | low | low | low | low | low | med | low | low | low | high |
| Farjam [49] | IJROBP | DCE | 3 | low | med | low | med | med | low | med | low | low | high | low | high |
| Wong [51] | Clin Canc Res | DCE | 3 | high | med | high | low | med | med | med | low | low | high | med | high |
| Gulaldi, [51] | ANM | SPECT | 1 | high | med | low | high | high | med | med | high | low | low | high | high |
| Hahn [34] | IJROBP | PET | 3 | med | low | low | high | med | high | med | med | high | high | high | high |
| Taki [32] | ANM | SPECT | 1 | high | high | low | med | high | high | med | med | low | high | high | high |
| Vera [35] | J Clin Oncol | SPECT | 3 | low | low | low | high | high | med | low | med | low | low | high | high |

**Supplementary Table 2.** Modified QUADAS-2 scoring for the metabolic articles.

|  |  |  | QUADAS D1 | | | | D2 | | | D3 | | D4 | D5 | | |
| --- | --- | --- | --- | --- | --- | --- | --- | --- | --- | --- | --- | --- | --- | --- | --- |
| Study | Journal | Acq | Study | Sample | Selection | Case | Acquisition | Analysis | ROI | Diagnosis | Treatment | Flow Timing | Exclusions | Statistics | Data |
| Alirezaei [79] | Biomed Res Int | 1H MRS | 3 | high | high | low | med | med | low | low | low | low | low | low | high |
| Chawla [76] | J Neuroimag | 1H MRS | 3 | low | med | low | low | low | low | med | low | low | high | low | high |
| Chernow [68] | The Neurorad J | 1H MRS | 3 | low | med | low | low | low | med | high | low | high | low | low | high |
| Davidson [83] | Med Pediatr Oncol | 1H MRS | 2 | high | high | high | med | med | med | high | high | high | low | med | high |
| Davidson [69] | BJR | 1H MRS | 4 | high | high | high | high | med | med | high | high | high | high | med | high |
| de Ruiter [86] | Human Brain Map | 1H MRS | 4 | low | low | low | med | med | low | med | low | low | low | med | high |
| Esteve [80] | IJ Rad Oncol Biol Phys | 1H MRS | 3 | high | med | low | low | low | low | low | med | low | low | low | high |
| Follin [84] | Psychoneuroendocrin | 1H MRS | 4 | low | low | high | med | med | low | high | med | low | low | low | high |
| Hattingen [89] | Neuro-Oncology | 1H MRS | 3 | low | low | low | low | low | med | low | low | low | low | low | high |
| Kaminaga [72] | JCAT | 1H MRS | 3 | low | low | low | med | low | low | med | low | low | low | low | high |
| Kesler [85] | Brain Imag Behav | 1H MRS | 2 | high | low | low | med | med | low | high | high | high | high | med | high |
| Lee [73] | JMRI | 1H MRS | 3 | low | med | high | med | med | low | high | med | low | high | med | high |
| Pospisil [77] | Rad Oncol | 1H MRS | 3 | low | low | low | low | low | low | low | high | high | low | low | high |
| Pospisil [78] | Rad Oncol | 1H MRS | 3 | low | low | low | low | low | low | low | high | high | low | low | high |
| Rutkowski [71] | IJ Rad Oncol Biol Phys | 1H MRS | 2 | high | high | high | low | low | low | high | med | high | high | med | high |
| Rueckriegel [74] | IJ Rad Oncol Biol Phys | 1H MRS | 2 | high | high | high | med | med | low | high | high | high | high | med | high |
| Stouten-Kemperman [87] | Brain Imag Behav | 1H MRS | 2 | low | low | low | med | med | high | med | low | high | low | med | high |
| Stadlbauer [46] | EJR | 1H MRS | 2 | low | med | high | low | low | low | med | low | low | low | low | high |
| Sundgren [81] | JMRI | 1H MRS | 3 | high | high | high | med | med | low | high | med | low | low | med | high |
| Tong [88] | Breast Cancer | 1H MRS | 4 | low | med | low | med | med | high | low | high | low | low | med | high |
| Usenius [70] | IJ Rad Oncol Biol Phys | 1H MRS | 2 | high | high | low | high | low | high | low | high | high | high | high | high |
| Virta [82] | MRI | 1H MRS | 2 | high | high | low | high | high | med | low | high | high | high | low | high |
| Waldrop [82] | AJNR | 1H MRS | 2 | high | low | high | med | med | low | med | high | high | low | med | high |
| Carideo [97] | BJR | PET | 1 | low | low | high | med | high | med | high | high | high | high | med | high |
| Hahn [34] | IJROBP | PET | 3 | med | low | low | high | med | high | med | med | high | high | high | high |
| Pomykala [90] | Brain Imag Behav | PET | 4 | low | low | low | med | med | med | high | high | low | low | med | high |
| Ponto [92] | Int J Geriatr Psych | PET | 2 | high | low | low | med | med | high | high | med | low | high | med | high |
| Schroyen [94] | Cancers | PET | 4 | low | low | low | low | med | med | low | low | low | low | low | high |
| Shrot [95] | Pediatric Neurology | PET | 1 | low | low | low | med | low | high | med | med | low | low | high | high |
| Silvernan [91] | Breast Cancer Res Treat | PET | 4 | high | high | low | med | high | high | high | high | high | med | high | high |
| Sorokin [96] | Clin Nuc Med | PET | 1 | high | high | high | high | med | high | high | high | high | high | high | high |
| Vitor [93] | An Nuc Med | SPECT | 4 | low | low | low | med | med | high | low | med | low | high | high | high |

**Supplementary Table 3.** Modified QUADAS-2 scoring for the advanced imaging articles.

|  |  |  | QUADAS D1 | | | | D2 | | | D3 | | D4 | D5 | | |
| --- | --- | --- | --- | --- | --- | --- | --- | --- | --- | --- | --- | --- | --- | --- | --- |
| Study | Journal | Acq | Study | Sample | Selection | Case | Acquisition | Analysis | ROI | Diagnosis | Treatment | Flow Timing | Exclusions | Statistics | Data |
| Chen [120] | Magn Reson Imag | QSM | 4 | high | low | low | med | low | med | med | med | low | low | low | high |
| Cushing [121] | Redox Biol | QSM | 3 | high | high | high | med | med | med | high | med | low | high | low | high |
| Mehrabian [122] | Nat Sci Rep | qMT | 3 | high | med | low | low | low | low | low | med | low | low | low | high |
| Cushing [121] | Redox Biol | T2* | 3 | high | high | high | med | low | low | high | med | low | high | low | high |
| Steen [123] | I J Rad Oncol Biol Phys | T1 | 3 | low | med | high | med | med | low | med | high | low | low | low | high |
| Billiet [115] | Brain Imag Behav | MWI, NODDI, DKI | 4 | high | low | low | low | low | low | med | low | low | low | low | high |
| Chakhoyan [114] | J NeuroOncol | DKI | 3 | low | med | high | med | med | low | med | low | high | high | low | high |
| Romero-Garcia [113] | J Neurosurg | NODDI | 3 | high | low | low | med | med | low | low | high | high | high | low | high |
| Sleurs [114] | Hum Brain Map | DKI, NODDI | 4 | low | low | low | low | med | low | med | med | low | low | low | High |
| Stouten-Kemperman [118] | Hum Brain Map | DKI | 2 | low | low | low | low | low | low | high | low | high | low | med | high |
| Tso [117] | Frontiers Oncol | DKI | 2 | low | low | low | med | med | low | low | low | low | low | low | high |
| Wu [116] | Clin Trans Rad Oncol | DKI | 3 | low | med | low | med | med | low | low | low | low | low | low | high |
| Wu [126] | J Oncol | DKI | 3 | low | med | low | med | Med | low | low | low | low | low | low | high |

**Modified Quadas-2 checklist**

Authors: Louise Hogeboom, Frederik Barkhof, Marek Chmelik, Patricia Clement, Sabine Deprez, Philip De Witt Hamer, Kyrre Eeg Emblem, Liviu-Andrei Fodor, Louise Hogeboom, Radim Jancalek, Jesper Kallehauge, Maarten Lequin, Vera C. Keil, Henk-Jan Mutsaerts, Ruben Emanuel Nechifor, Pavel Nikulin, Jan Petr, Cyril Pernet, Francesca Pizzini, Gwen Schroyen, Evita Wiegers

# **Original QUADAS 2 checklist**

**(Developed by a consortium of researchers from the UK, the US, and the NL) can be found here:** [Quadas2](https://www.bristol.ac.uk/population-health-sciences/projects/quadas/quadas-2/) or here: [QUADAS-2: A Revised Tool for the Quality Assessment of Diagnostic Accuracy Studies | Annals of Internal Medicine (acpjournals.org)](https://www.acpjournals.org/doi/full/10.7326/0003-4819-155-8-201110180-00009).

It is used to evaluate the quality of literature sources added to systematic reviews and has 4 domains (perspectives of analysis of the sources):

- patient selection
- index test
- reference standard
- flow and timing

Each domain assesses the study regarding the risk of bias in the study results and the first three domains also rate applicability. For the purpose of the review on adverse effects of cancer treatment, we modified the domains in some aspects, and added a fifth one, to adapt them better to medical imaging studies. This modification is denoted the **“modified QUADAS-2“** version.

# **Domains explained**

When you work with this *modified QUADAS-2*, for each domain below you need to screen the paper for a) SIGNALLING TEXT (=crucial bits of information) and b) METHODOLOGY (Can the study be reproduced based on the information given?, and c) ADEQUATENESS (are the methods described in the study adequate to answer the research question?)

## **Domain 1: Patient selection**

1. **Study design (Study design: label 1-4)**
   - states the risk of bias: retrospective versus prospective articles, randomized controlled trials versus case-controlled studies
   - *EXAMPLE: “This was a single-center, retrospective study approved by the Institutional Review Board, and was Health Insurance Portability and Accountability Act compliant.”*
   - **SCORING**:
     - **4**: prospective, matched (healthy) controlled trials
     - **3**: prospective, non-matched controls or no controls
     - **2**: retrospective, matched or otherwise balanced
     - **1**: retrospective, no matching or balancing of groups stated
2. **Was the patient selection unbiased - consecutive or random sampling of patients was done (Sample selection: Low/high risk of bias)**
   - **SCORING:**
     - **Low risk:** Consecutive sampling - all eligible patients from a well-defined period were included. Random sampling - randomly selected patients were included.
     - **High risk:** No consecutive or random sampling or the description is unclear or missing important details.
3. **Are selection criteria clearly described? (Selection criteria: low/medium/high risk of bias)**
   - Were all relevant inclusion and exclusion criteria described in the method section?
   - **EXAMPLES:**
     - Good, low risk of bias: “*Inclusion criteria consisted of the pathological diagnosis of GBM with subsequent tumor recurrence, treatment with BEV and pcASL MRI sequence acquisition performed before and/or during BEV treatment. All subjects underwent standard of care clinical workup consisting of resection and/or biopsy to establish a pathological diagnosis of GBM. An initial Stupp protocol was followed for all subjects consisting of radiotherapy (XRT) administered in conjunction with temozolomide (TMZ). All subjects included in this study were diagnosed with recurrent GBM based upon clinical and/or conventional radiological data interpreted by a neuro-oncologist, based upon current RANO criteria. Pseudoprogression was not observed in any of the included cases. ” (Andre et al. 2015)*
     - *Medium* risk of bias: “*A retrospective review of pathology reports from patients with suspected recurrent or progressive HGG (World Health Organization Grade III or IV) including anaplastic astrocytoma and glioblastoma diagnosed at University of Colorado Hospital from 2011 to 2014 was performed. Patients were included if they had previously been treated with chemotherapy, radiation, and surgery according to the standard of care, and later underwent biopsy or re-resection during follow-up for suspected disease progression, and had diagnostic ASL imaging performed on a single 3 T MRI scanner prior to biopsy. None of the patients in this study had been treated with anti-angiogenic agents”.* (Nyberg et al. 2016)
       - **While the last example *seems* complete, many questions remain open. What chemotherapy exactly? What is the standard of care (in the hospital? National? Global?)? Check where the authors are VAGUE.**
   - **SCORING:**
     - **low risk of bias**: all relevant criteria mentioned clearly
     - **medium risk of bias**: key criteria mentioned with some doubts for the details
     - **high risk of bias**: key criteria are very vague or missing
4. **Case description (case description: low/high risk of bias)**
   - Information on participants complete. Demographics (age - mean+SD or range, male/female ratio, primary disease).
   - **SCORING**
     - **low risk of bias:** information is complete for all patients and also for all separately examined subgroups.
     - **high risk of bias:** important information is missing, unclear, or incomplete.

## **Domain 2: Index test**

1. **Index test ranking for the imaging acquisition (Acquisition - points + low/medium/high risk of bias)**
   - Was the imaging described in all details, all the necessary parameters are given, and all necessary processing steps are done and described. For this question concerning the applicability, it must be checked if certain keyword items were specified that are needed for reproduction and for a good quality of quantification.
   - A ranking guide for each imaging technique is at the bottom of this document. Perform for each imaging technique used independently.
   - **Use the tables for different MRI sequences that are provided at the end of the document**.
   - **The sum can be provided with cut-off values to separate/distinguish good from badly explained methodology.**
   - **SCORING:**
     - **Report both the final points and the risk of bias level**
     - **low risk**: 12-15 points in the index test
     - **medium risk**: 7-11 points in the index test
     - **high risk**: 0-6 points in the index test
2. **Image analysis described in detail (image analysis - low/medium/high risk of bias)**
   - Do the authors provide enough details of the image analysis? This includes the description of image pre-processing, quantification of the imaging in question, and joint processing and alignment of the multimodal data (structural and quantitative images and, if available, RT treatment planning scans and RT dose maps)
   - Alternatively, full data analysis scripts are publicly available and details about their use are given.
   - Alternatively, publicly available software was used for processing and parameter settings were clearly described.
   - Alternatively, a reference to a prior publication that contains the details.
   - **SCORING:**
     - **low risk**: a full account of the used methods is described
     - **medium risk**: important details in methods used or settings are missing
     - **high risk**: image processing description is missing or is unclear or vague
3. **Region of interest (ROI) and data extraction (ROI - low/medium/high risk of bias)**
   - Do the authors provide sufficient details on where and how the quantitative imaging parameters were extracted? It either has to be explained how the ROIs were manually delineated or details about an automatic procedure need to be provided. And the authors need to explain how the data were extracted from these ROIs.
   - **SCORING:**
     - **low risk**: all details about the ROIs and data extraction is given
     - **medium risk**: ROIs are described, but important details are missing in how these were delineated or on how the data were extracted
     - **high risk**: ROI definition is too vague, unclear, or missing

## **Domain 3: Reference test**

1. **Was the tumor classification done according to the state-of-the-art? (Diagnosis low/high risk of bias)**
   - Here, you assess if a tumor diagnosis was done to the clinical standards including the relevant imaging, histopathology, and molecular subtyping.
   - This concerns the tumor diagnosis, not the normal tissue measurements.
   - **Specifically for gliomas**: Glioma tumor subgrades should be classified according to the old WHO (pre-2016) grading system, or ideally, according to the revised 2016 WHO grading system (Louis et al. 2016). The latter information may be hard to come by, however, especially for older studies, but at a minimum IDH status is of high relevance for the new classification. In the ideal case, this should also include diffuse midline glioma status, 1p/19q codeletion, and TP53. For higher grade gliomas (~glioblastoma), MGMT promoter methylation status is also relevant because of its sensitivity to chemotherapy (MGMT met = longer overall survival) *(Hegi et al. 2005)*  and the concept that a glioma/glioblastoma is a systemic (whole-brain) disease also affecting surrounding, normal-appearing tissue.
   - **SCORING:**
     - **low risk:** all details were reported
     - **medium risk**: missing details about tumor subclassification (e.g. MGMT)
     - **high risk**: missing important details about tumor grade or subgrade (e.g. IDH status for studies done after 2016)
2. **Are all details about the treatment provided? (Treatment low/medium/high risk)**
   - Was the type and details about the treatment described for all patients.
   - Alternatively, is previous work or relevant article describing the clinical standard used cited.
   - Was the extent of surgery, or biopsy only, described for all patients.
   - **Radiotherapy:** is the type given (WBRT, RT, SRS), beam type and planning (Photon/Proton/IMRT/3DCRT), Clinical target volume delineation, Device manufacturer, Total dose in the target volume, daily fraction or number of fractions described?
   - **Chemotherapy:** are the type, dose, and duration described. Ideally the number of cycles, length of cycle, and daily dose?
   - **Other drugs**: relevant for the study as a potential source of adverse effects (e.g. immunotherapy, hormone therapy): is the type, dosage and duration described?
   - **Previous cancer treatment:** (for recurrent or progressing disease) is mentioned and briefly described, or part of inclusion/exclusion criteria?
   - **SCORING:**
     - **low risk:** all relevant information is given for all treatment types included in the study
     - **medium risk:** minor details are missing for one or more treatment types
     - **high risk:** important information is missing for one or more treatment types

## **Domain 4: Flow and timing**

1. **Is the timing of the treatment and imaging well explained? (Flow timing low/high risk of bias)**
   - Is the timeline of the experimental setup well described. The authors need to properly describe when were different components of the treatment (surgery, radiochemotherapy, other drugs) applied relative to each other. And when was the baseline and follow-up imaging session performed with respect to the treatment?
   - **SCORING**
     - **low risk:** all relative timings described
     - **high risk:** unclear how are different parts of the treatment relative to each other in time or when, relative in time to the treatment, was the imaging done - concerning both the baseline and all the follow-ups.

## **Domain 5: Data analysis, processing and reporting**

1. **Were withdrawals from the study explained and uninterpretable results reported? (Exclusions: low/high risk)**
   - Do the authors give reasons why subjects and scans are excluded from the study and properly list the numbers of exclusions.
   - This question addresses both subject-level exclusions and also exclusions due to low data quality or missing measurements.
   - Example, low risk of bias*: “We exclude 33 patients in whom no newly appeared or enlarged enhancing lesions were detected, 5 patients without the final diagnosis and 3 patients with severe motion artifacts affecting the visualization of perfusion imaging.”* (Razek et al. 2018). Prospective study - excluding subjects as no longer fulfill inclusion criteria or have low-quality data. Reasons and numbers are properly described.
   - Example, High risk of bias: “*The data would be excluded if they were of poor quality”. (Lyu et al. 2017)* Failure to explain what low quality means or report the number of excluded subjects/scans.
   - **SCORING:**
     - **low risk: a** mechanism for exclusion is clearly explained and the number of excluded subjects/scans is given
     - **high risk:** data on non-participating subjects and excluded scans are incomplete or missing, an unexplained mismatch between the number of recruited subjects, examined scans, and reported results.
2. **Are the statistical tests sufficient, reported, and correct? (Statistical tests: low/medium/high risk of bias)**
   - Are significant AND non-significant results stated with p-values?
   - Are confidence intervals and effect sizes stated?
   - Are all statistical tests used mentioned?
   - If the main study finding is that no significant results are present, then a power calculation is done to show that the study cohort is large enough
   - Controlling for confounding factors (if relevant)
   - Use of correction for multiple comparisons (if relevant - multiple tests were used)
   - **SCORING:**
     - **low risk**: All of the above is mentioned and considered
     - **medium risk**: Most of the above-mentioned, missing power calculation, confounders, multiple comparison correction.
     - **high risk**: Statistics part has clear gaps of information hampering reproduction of results OR used tests not fitting the research question OR missing key values like p-values or effect sizes
3. **Data availability (Data: low/high risk of bias)**
   - Do authors make the raw data publicly available OR
   - Data from a public database were used OR
   - Derived data (i.e. key quantitative results per subject) are available online or in the supplementary material
   - Note that this is not the case for most publications, but the incentive is to do this in the future.
   - **SCORING:**
     - **low risk:** Data are available in some form
     - **high risk:** Raw data nor derivatives are not available in any form

## **15 point list KEYWORD INDEX TEST:**

Scores up to 15 points for each modality. The first 7 points are common for all modalities, the remaining 8 are sequence-specific.

| **ALL IMAGING STUDIES** | One point per criterion |
| --- | --- |
| 1. Scanner type and manufacturer (MRI: Field strength) 2. MR head coil type (SPECT: used collimator) 3. Resolution and FOV: in-plane FOV/resolution, slice thickness/axial FOV OR voxel size 4. MRI: TR, TE, Flip angle 5. Scan duration (and framing scheme, if applicable) 6. Software for postprocessing mentioned 7. Registration, segmentation/ROI drawing, etc. method explained |  |
| **DSC** |  |
| 1. Sequence type; gradient-echo or spin-echo 2. Contrast agent name and concentration 3. Flow rate of injection 4. Presaturation T1 with gadolinium before dynamic injection 5. Number of repetitions/dynamic scans, Time per dynamic 6. Choice of the arterial input function 7. Leakage correction 8. Normalization to reference tissue, including what type of reference tissue (gray and/or white matter) |  |
| **DCE** |  |
| 1. Contrast agent name, concentration, saline flush reported 2. Flow rate of injection, hand or power injector, rate >2mL/s 3. Baseline T1 determined, signal to concentration performed, baseline S0>=5 4. Number of repetitions/dynamic scans, total time > 5min 5. Time per dynamic reported and < 3s 6. Choice of arterial input function - measured, population, none 7. Analysis model, e.g. Tofts 8. Parameters generated (K^trans^, V_p_, V_e_...) |  |
| **ASL** |  |
| 1. Post-labeling delay 2. Labeling duration 3. Number of excitations (NEX) or control/label pairs 4. Some form of M0 calibration used 5. Background suppression described 6. Labeling and readout type is given (e.g. pCASL 3DGRASE) 7. Exclusion of low-quality scans (long ATT, motion, failed labeling) 8. Labeling plane position |  |
| **DKI** |  |
| 1. Sequence type 2. How many diffusion encoding directions? Minimum requirements: at least 3 b values around [0 1000 2000] and at least 14 dir in each shell to determine the 4 rank tensor 3. Which b-values were used 4. Gibbs ringing is corrected 5. Number of signal averages 6. Parameter estimation (e.g. LLS, NLLS, or sequential LLS->NLLS) 7. Correction for gradient inhomogeneities 8. Denoising performed |  |
| **PET** |  |
| 1. Used tracer 2. Dosage 3. Post-injection scan delay 4. Patient conditions (for FDG: eyes/ears occlusion, fasting, waiting room conditions) 5. Acquisition specifics (TOF/non-TOF, 2D/3D if applicable for the given scanner) 6. Reconstruction details (iterative: number of subsets/iterations, regularizations and/or PSF modeling (if available); FBP: used filter) 7. Attenuation (type), scatter, randoms corrections 8. PET quantification (method for derivation of absolute values) |  |
| **SPECT** |  |
| 1. Used tracer 2. Dosage 3. Post-injection scan delay 4. Number of heads 5. Number of acquisition angles and total span 6. Reconstruction details (iterative: number of subsets/iterations; FBP: used filter) 7. Spatial resolution stated and plausible 8. SPECT quantification (attenuation correction; rCBF derivation method/normalization reference region) |  |
| **MRS** |  |
| 1. Sequence type (Single voxel (PRESS, STEAM) or MRSI (2D,3D)) 2. SVS or MRSI position selection 3. Number of signal averages 4. Shimming method and average water linewidth 5. Baseline correction 6. Fitting model used (e.g. LCmodel) and prior knowledge description 7. Relative (specify reference metabolite) or absolute concentrations 8. Cramér Rao Lower Bounds (CRLB) |  |
| **QSM** |  |
| 1. QSM acquisition method explained at all 2. R2* acquistion method explained at all 3. Filtering explained general 4. Background field removal explained 5. QSM reconstruction algoritnm explained (2 points) 6. Normalization for measurements explained (2 points) |  |
| **qMT** |  |
| 1. Power amplitudes spectrum mentioned 2. Offset frequencies mentioned 3. Saturation durations mentioned 4. B1 and R1 mapping method mentioned 5. T2 mapping method mentioned 6. MT model mentioned 7. Model equations mentioned 8. Normalization of MT spectrum technique mentioned |  |
| **NODDI** |  |
| 1. e gradient pulse width δ OR gradient directions named 2. b-values named 3. gradient strength variation 4. assumed intrinsic/isotropic diffusivity named 5. Fitting procedure described 6. NODDI model stated 7. DWI sequence type mentioned 8. Maps generated mentioned |  |
| **Relaxometry** |  |
| 1. Sequence type (T1, T2, PD, T1+C, T1 subtraction maps, FLAIR, 3D T1 MPRAGE, 3D/SPACE acquisition) (2 points) 2. Number of signal averages (2 points) 3. Image reconstruction/fitting technique (2points) 4. Image orientation (Ax, Sag, Cor) (2 points) |  |

## **References:**

Andre JB, Nagpal S, Hippe DS, Ravanpay AC, Schmiedeskamp H, Bammer R, et al. Cerebral Blood Flow Changes in Glioblastoma Patients Undergoing Bevacizumab Treatment Are Seen in Both Tumor and Normal Brain. Neuroradiol J. 2015 Apr;28(2):112–9.

Hegi ME, Diserens AC, Gorlia T, Hamou MF, de Tribolet N, Weller M, Kros JM, Hainfellner JA, Mason W, Mariani L, Bromberg JE, Hau P, Mirimanoff RO, Cairncross JG, Janzer RC, Stupp R. MGMT gene silencing and benefit from temozolomide in glioblastoma. N Engl J Med. 2005 Mar 10;352(10):997-1003. doi: 10.1056/NEJMoa043331. PMID: 15758010.

Louis DN, Perry A, Reifenberger G, von Deimling A, Figarella-Branger D, Cavenee WK, Ohgaki H, Wiestler OD, Kleihues P, Ellison DW. The 2016 World Health Organization Classification of Tumors of the Central Nervous System: a summary. Acta Neuropathol. 2016 Jun;131(6):803-20

Lyu Y, Liu S, You H, Hou B, Wang Y, Ma W, et al. Evaluation of recurrent high-grade gliomas treated with bevacizumab: A preliminary report of 3D pseudocontinuous artery spin labeling. J Magn Reson Imaging. 2017 Aug;46(2):565–73.

Nyberg E, Honce J, Kleinschmidt-DeMasters BK, Shukri B, Kreidler S, Nagae L. Arterial spin labeling: Pathologically proven superiority over conventional MRI for detection of high-grade glioma progression after treatment. Neuroradiol J. 2016 Oct;29(5):377–83.

Razek AAKA, El-Serougy L, Abdelsalam M, Gaballa G, Talaat M. Differentiation of residual/recurrent gliomas from postradiation necrosis with arterial spin labeling and diffusion tensor magnetic resonance imaging-derived metrics. Neuroradiology. 2018 Feb;60(2):169–77.

##

## Search term for Web of Science

(TI=(glioma* OR “glial tumor*” OR astrocytoma* or glioblastoma* OR GBM OR “oligodendroglial tumor*” OR oligodendroglioma* OR “brain metastasis” OR metastases OR “cns tumor*” OR “brain tumor*” OR “skull base tumor*” OR “primary CNS lymphoma*” OR PCNSL OR medulloblastoma* OR ependymoma* OR DIPG OR “diffuse midline” OR “pons glioma*” OR teratoma* OR pinealoblastoma* OR pineoblastoma* OR “germ cell tumor*” OR pilocytic OR SEGA OR “nasopharyngeal carcinoma*” OR craniopharyngioma* OR “Squamous Cell Carcinoma*” OR tumor* OR cancer* OR leukemia* OR “brain malignancy*” OR “brain RT” OR “brain radiation”) OR AB=(glioma* OR “glial tumor*” OR astrocytoma* or glioblastoma* OR GBM OR “oligodendroglial tumor*” OR oligodendroglioma* OR “brain metastasis” OR metastases OR “cns tumor*” OR “brain tumor*” OR “skull base tumor*” OR “primary CNS lymphoma*” OR PCNSL OR medulloblastoma* OR ependymoma* OR DIPG OR “diffuse midline” OR “pons glioma*” OR teratoma* OR pinealoblastoma* OR pineoblastoma* OR “germ cell tumor*” OR pilocytic OR SEGA OR “nasopharyngeal carcinoma*” OR craniopharyngioma* OR “Squamous Cell Carcinoma*” OR tumor* OR cancer* OR leukemia* OR “brain malignancy*” OR “brain RT” OR “brain radiation”))

AND

(TI=(“healthy brain*” OR “normal appearing brain*” OR “normal brain*” OR “normal appearing brainstem*” OR “non affected brain*” OR “adverse effect*” OR “post therapy effect*” OR “therapy related effect*” OR “radiation dose effect*” OR “normal tissue response” OR “detrimental effects of radiotherapy” OR neurotoxic* OR ((neurocognitive OR cognitive OR neurobehavioral) AND (damage OR decline OR impairment OR “function change” OR deterioration OR dysfunction)) OR “neurocognitive outcome*” OR “neurocognitive results” OR “cognitive function” OR “neurobehavioral performance” OR “language decline”) OR AB=(“healthy brain*” OR “normal appearing brain*” OR “normal brain*” OR “normal appearing brainstem*” OR “non affected brain*” OR “adverse effect*” OR “post therapy effect*” OR “therapy related effect*” OR “radiation dose effect*” OR “normal tissue response” OR “detrimental effects of radiotherapy” OR neurotoxic* OR ((neurocognitive OR cognitive OR neurobehavioral) AND (damage OR decline OR impairment OR “function change” OR deterioration OR dysfunction)) OR “neurocognitive outcome*” OR “neurocognitive results” OR “cognitive function” OR “neurobehavioral performance” OR “language decline”))

AND

(TI=(Neuroimaging OR VASO OR DOPA OR TRODAT OR TSPO OR VERDICT OR Perfusion OR DCE OR “dynamic contrast enhanced” OR DSC OR “dynamic susceptibility contrast OR ASL OR “arterial spin labeling” OR “arterial spin labelling” OR “CT perfusion” OR IVIM OR “intravoxel incoherent motion” OR “15O H2O” OR “15O water” OR “O PET” OR PWI OR “perfusion weighted imaging” OR microperfusion OR CEST OR APT OR “amide proton transfer” OR “chemical exchange saturation” OR “magnetisation transfer” OR MT OR “magnetization transfer” OR MRSI OR MRS OR spectroscopy OR relaxometry OR “quantitative MRI” OR qMRI OR QSM OR “quantitative susceptibility mapping” OR (scintigraphy AND hmpao) OR “Diffusion Kurtosis Imaging” OR “Myelin Water Imaging” OR “mcDESPOT” OR “NODDI”) OR AB=(Neuroimaging OR VASO OR DOPA OR TRODAT OR TSPO OR VERDICT OR Perfusion OR DCE OR “dynamic contrast enhanced” OR DSC OR “dynamic susceptibility contrast OR ASL OR “arterial spin labeling” OR “arterial spin labelling” OR “CT perfusion” OR IVIM OR “intravoxel incoherent motion” OR “15O H2O” OR “15O water” OR “O PET” OR PWI OR “perfusion weighted imaging” OR microperfusion OR CEST OR APT OR “amide proton transfer” OR “chemical exchange saturation” OR “magnetisation transfer” OR MT OR “magnetization transfer” OR MRSI OR MRS OR spectroscopy OR relaxometry OR “quantitative MRI” OR qMRI OR QSM OR “quantitative susceptibility mapping” OR (scintigraphy AND hmpao) OR “Diffusion Kurtosis Imaging” OR “Myelin Water Imaging” OR “mcDESPOT” OR “NODDI”))

AND

(TI=(“Cranial Radiation” OR radiotherapy OR “radiation induced” OR RT OR CRT OR RCT OR chemotherapy OR radiosurgery OR “whole brain radiation” OR radiochemotherapy OR chemoradiotherapy OR chemoradiation OR temozolomide OR “photon therapy” OR “photon radiation” OR “proton radiation” OR “proton therapy” OR (survivor* AND cancer) OR tumor* OR avastin OR bevacizumab OR “hormone therapy” OR ((antibody OR antibodies OR cytokine*) AND (therapy OR treatment)) OR immunotherapy) OR AB=(“Cranial Radiation” OR radiotherapy OR “radiation induced” OR RT OR CRT OR RCT OR chemotherapy OR radiosurgery OR “whole brain radiation” OR radiochemotherapy OR chemoradiotherapy OR chemoradiation OR temozolomide OR “photon therapy” OR “photon radiation” OR “proton radiation” OR “proton therapy” OR (survivor* AND cancer) OR tumor* OR avastin OR bevacizumab OR “hormone therapy” OR ((antibody OR antibodies OR cytokine*) AND (therapy OR treatment)) OR immunotherapy))

##

## Search term for PubMed

((glioma*[Title/Abstract]) OR (glioma*[Text word]) OR (glial tumor*[Title/Abstract]) OR (astrocytoma*[Title/Abstract]) OR (glioblastoma*[Title/Abstract]) OR (GBM[Title/Abstract]) OR (oligodendroglial tumor*[Title/Abstract]) OR (oligodendroglioma*[Title/Abstract]) OR (brain metastasis[Title/Abstract]) OR (metastases[Title/Abstract]) OR (cns tumor*[Title/Abstract]) OR (brain tumor*[Title/Abstract]) OR (skull base tumor*[Title/Abstract]) OR (primary CNS lymphoma*[Title/Abstract]) OR (PCNSL[Title/Abstract]) OR (medulloblastoma*[Title/Abstract]) OR (ependymoma*[Title/Abstract]) OR (DIPG[Title/Abstract]) OR (diffuse midline[Title/Abstract]) OR (pons glioma[Title/Abstract]) OR (teratoma[Title/Abstract]) OR (pinealoblastoma[Title/Abstract]) OR (pineoblastoma[Title/Abstract]) OR (germ cell tumor[Title/Abstract]) OR (pilocytic[Title/Abstract]) OR (SEGA[Title/Abstract]) OR (nasopharyngeal carcinoma*[Title/Abstract]) OR (craniopharyngioma*[Title/Abstract]) OR (Squamous Cell Carcinoma*[Title/Abstract]) OR (tumor*[Title/Abstract]) OR (cancer*[Title/Abstract]) OR (leukemia*[Title/Abstract]) OR (brain malignancy*[Title/Abstract]) OR (brain RT[Title/Abstract]) OR (brain radiation[Title/Abstract]))

AND ((healthy brain*[Title/Abstract]) OR (normal appearing brain*[Title/Abstract]) OR (normal brain*[Title/Abstract]) OR (normal-appearing white matter[Title/Abstract]) OR (normal appearing brainstem*[Title/Abstract]) OR (non affected brain*[Title/Abstract]) OR (adverse effect*[Title/Abstract]) OR (post therapy effect*[Title/Abstract]) OR (therapy related effect*[Title/Abstract]) OR (radiation dose-effects[Title/Abstract]) OR (normal tissue response[Title/Abstract]) OR (detrimental effects of radiotherapy[Title/Abstract]) OR (neurotoxic*[Title/Abstract]) OR ( ((neurocognitive[Title/Abstract]) OR (cognitive[Title/Abstract]) OR (neurobehavioral[Title/Abstract])) AND ((damage[Title/Abstract]) OR (decline[Title/Abstract]) OR (impairment[Title/Abstract]) OR (function change[Title/Abstract]) OR (deterioration[Title/Abstract]) OR (dysfunction[Title/Abstract])) ) OR (“neurocognitive outcomes”[Title/Abstract]) OR (“neurocognitive results”[Title/Abstract]) OR (“*cognitive function*”[Title/Abstract]) OR (“neurobehavioral performance”[Title/Abstract]) OR (“language decline”[Title/Abstract]))

AND ((Neuroimaging[Title/Abstract]) OR (VASO[Title/Abstract]) OR (DOPA[Title/Abstract]) OR (TRODAT[Title/Abstract]) OR (TSPO[Title/Abstract]) OR (VERDICT[Title/Abstract]) OR (Perfusion[Title/Abstract]) OR (DCE[Title/Abstract]) OR (dynamic contrast enhanced[Title/Abstract]) OR (DSC[Title/Abstract]) OR (dynamic susceptibility contrast[Title/Abstract]) OR (ASL[Title/Abstract]) OR (arterial spin labeling[Title/Abstract]) OR (arterial spin labelling[Title/Abstract]) OR (CT perfusion[Title/Abstract]) OR (IVIM[Title/Abstract]) OR (intravoxel incoherent motion[Title/Abstract]) OR (15O H2O[Title/Abstract]) OR (15O water[Title/Abstract]) OR ('O PET'[Title/Abstract]) OR (PWI[Title/Abstract]) OR (perfusion-weighted imaging[Title/Abstract]) OR (microperfusion[Title/Abstract]) OR (CEST[Title/Abstract]) OR (APT[Title/Abstract]) OR (amide proton transfer[Title/Abstract]) OR (chemical exchange saturation[Title/Abstract]) OR (magnetisation transfer[Title/Abstract]) OR (MT[Title/Abstract]) OR (magnetization transfer[Title/Abstract]) OR (MRSI[Title/Abstract]) OR (MRS[Title/Abstract]) OR (spectroscopic[Title/Abstract]) OR (spectroscopy[Title/Abstract]) OR (relaxometry[Title/Abstract]) OR (quantitative MRI[Title/Abstract]) OR (qMRI[Title/Abstract]) OR (QSM[Title/Abstract]) OR (quantitative susceptibility mapping[Title/Abstract]) OR (scintigraphy[Title/Abstract] AND hmpao[Title/Abstract]) OR (Diffusion Kurtosis Imaging[Title/Abstract]) OR (Myelin Water Imaging[Title/Abstract]) OR (mcDESPOT[Title/Abstract]) OR (NODDI[Title/Abstract]))

AND ((Cranial Radiation[Title/Abstract]) OR (radiotherapy[Title/Abstract]) OR (radiation induced[Title/Abstract]) OR (RT[Title/Abstract]) OR (CRT[Title/Abstract]) OR (RCT[Title/Abstract]) OR (chemotherapy[Title/Abstract]) OR (radiosurgery[Title/Abstract]) OR (whole brain radiation[Title/Abstract]) OR (radiochemotherapy[Title/Abstract]) OR (chemoradiotherapy[Title/Abstract]) OR (chemoradiation[Title/Abstract]) OR (temozolomide[Title/Abstract]) OR (photon therapy[Title/Abstract]) OR (photon radiation[Title/Abstract]) OR (proton radiation[Title/Abstract]) OR (proton therapy[Title/Abstract]) OR ((survivor*[Title/Abstract]) AND ((cancer[Title/Abstract]) OR (tumor*[Title/Abstract]))) OR

avastin[Title/Abstract] OR bevacizumab[Title/Abstract] OR ((antibody[Title/Abstract] OR antibodies[Title/Abstract] OR cytokine*[Title/Abstract]) AND (therapy[Title/Abstract] OR treatment[Title/Abstract])) OR immunotherapy[Title/Abstract] OR (hormone therapy[Title/Abstract]))
