# Supplementary material for: A systematic review on the use of quantitative imaging to detect cancer therapy adverse effects in normal-appearing brain tissue
Source: MAGMA. 2021 Dec 17;35(1):163–86. doi: 10.1007/s10334-021-00985-2 (PMC8901489; doi:10.1007/s10334-021-00985-2)
Supplement: Supplementary file 2 — Supplementary file2 (DOCX 24 KB) [file 10334_2021_985_MOESM2_ESM.docx]

**Research protocol**

1. **Define the research topic, the research question, and establish the PICO’S protocol.**

| **Research Topic** | **Research Question** | **PICO** | |
| --- | --- | --- | --- |
| A systematic review of detectability of cancer therapy effects on normal-appearing brain tissue using quantitative imaging  **CNS =** Central Nervous System | What is the development trajectory of the neuroimaging detected changes in neurocognitive function in patients with neoplasm?  **Type of question:** Etiology/Harm | **Population** | Patients of all ages and sexes. |
|  |  | **Intervention/**  **Exposure** | Exposed to cancer-related systemic treatments. No restrictions were considered for the radiotherapy/chemotherapy/immunotherapy regimen/protocol. |
|  |  | **Comparisons** | With pre-therapy baseline or control group without therapy. |
|  |  | **Outcome** | Change in treatment-related cognitive impairment or brain metabolism/function/physiology changes measured by neuroimaging techniques. |
|  |  | **Study design** | Longitudinal and cross-sectional prospective and retrospective studies. |

1. **Define the main search terms, based on PICO’S.**

| **Neoplasm** | **Normal tissue effects** | **Neuroimaging** | **Cancer therapy** |
| --- | --- | --- | --- |

1. **Define the search terms based on the previous steps.**

| Keywords Identification | | |
| --- | --- | --- |
|  | **1. Neoplasm** | Breast cancer, neoplasm, glioma, metastasis, tumor, leukemia, cancer, etc. |
|  | **2. Normal tissue effects** | Cognitive decline, normal brain tissue, adverse effects, etc. |
|  | **3. Neuroimaging** | Neuroimaging, PET/SPECT (also including DOPA, FDG, TRODAT, HMPAO, TSPO, 15O-H2O, water), perfusion, ASL, DSC, DCE, IVIM, MT, MRSI, MRS, spectroscopy, relaxometry, qMRI, QSM, VASO, DKI, MWI, mcDESPOT, NODDI, VERDICT, CEST, etc. |
|  | **4. Cancer therapy** | Radiotherapy, hormone therapy, chemotherapy, chemoradiation, immunotherapy, etc. |

1. **Search for papers on the databases.**

MEDLINE and Web of Science.

1. **Document all search procedures for each database.**

A full account of the search terms is provided in Supplementary Materials.

1. **Establishment of the eligibility criteria.**

| **Eligibility** **criteria** | | |
| --- | --- | --- |
|  | **Inclusion Criteria** | **Exclusion criteria** |
| **Type of participants** | - Human patients of all ages and sexes with past or current neoplasia | - Animal, preclinical, or phantom studies |
| **Type of interventions** | - Exposed to local or systemic cancer-related treatment by radio/chemo/immuno/hormone therapy |  |
| **Type of study /design** | - Longitudinal retrospective or prospective studies, with a baseline measure (before systemic treatment) - Cross-sectional studies with a control group without treatment - Normal-brain-tissue damage assessed by quantitative neuroimaging techniques. - Published in a peer-reviewed journal | - Cross-sectional studies without a control group - Longitudinal studies without a pre-therapy measurement - Not available in English - Not published in a peer-reviewed journal - Other reviews, conference proceedings, case studies, protocols |
| **Type of outcome** | - Cancer-related cognitive impairment measured with neuroimaging techniques | - Structural MRI studies including - VBM, cortical thickness, GM volume, DTI (FA, tractography), DWI (ADC), SWI/STI, fMRI, structural networks, default mode networks |

1. **Risk of bias assessment.**

Assessed using a modified-QUADAS-2 tool. Details are provided in the Supplementary Materials.
